# Cholesterol-Functionalized Porous PLA Microparticles for Enhanced Drug Delivery

**DOI:** 10.1021/acsabm.5c00693

**Published:** 2025-09-04

**Authors:** Ahammed HM Mohammed-Sadhakathullah, Leonor Resina, Hamidreza Enshaei, Kristina Ivanova, Tzanko Tzanov, Maria M. Pérez-Madrigal, Elaine Armelin, Juan Torras

**Affiliations:** † Innovation in Materials and Molecular Engineering − Biomaterials for Regenerative Therapies (IMEM-BRT) Group, Departament d’Enginyeria Química, EEBE, 16767 Universitat Politècnica de Catalunya, C/Eduard Maristany 10-14, Building I, second floor, Barcelona 08019, Spain; ‡ Barcelona Research Center for Multiscale Science and Engineering (BRCMSE), Universitat Politècnica de Catalunya, C/Eduard Maristany, 10-14, Building I, basement floor, Barcelona 08019, Spain; § Grup de Biotecnologia Molecular i Industrial, Department of Chemical Engineering, 16767 Universitat Politècnica de Catalunya, Rambla Sant Nebridi 22, Terrassa 08222, Spain

**Keywords:** poly(lactic acid) microparticles, poly(ethylene glycol)−cholesterol, curcumin, Tamoxifen, breast cancer therapy

## Abstract

A drug delivery platform based on highly porous poly­(lactic acid) (PLA) microparticles functionalized with amphiphilic poly­(ethylene glycol)–cholesterol (PEG–Chol) has been developed and successfully validated *in vitro*. This hybrid system addresses key limitations of conventional PLA and poly­(lactide-*co*-glycolide) (PLGA) nanoparticles, providing better encapsulation and sustained drug release. The incorporation of PEG–Chol provides both enhanced aqueous dispersibility for prolonged circulation and membrane-anchoring capabilities, thereby promoting cellular interaction and endocytosis. The particles were loaded with two lipophilic anticancer agents, curcumin (Cur) and tamoxifen (Tmx), whose clinical use is constrained by poor solubility and systemic side effects. In vitro studies using MCF-7 breast cancer cells demonstrated successful cellular uptake and significantly reduced cell viability, validating the therapeutic potential of the system. These results highlight the promise of lipid-functionalized porous PLA particles as a versatile and effective platform for advanced drug delivery in breast cancer treatment.

## Graphical abstract

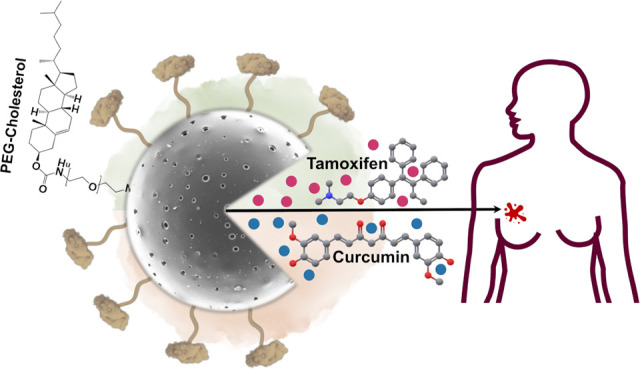

## 1 Introduction

Micro (MPs)- and nanoparticles (NPs)-based on poly­(lactic acid) (PLA) and poly­(lactide-*co*-glycolide) (PLGA) are widely used as drug delivery systems,^1,2^ but their hydrophobic nature limits the sustained drug release being quickly removed from circulation after parenteral administration. Though smaller particles improve drug delivery efficiency, PLGA particles under 10 μm are easily phagocytosed by immune cells.^3^ To address these challenges, strategies such as PEGylation, copolymerization with hydrophilic polymers, and coating with surfactants or targeting ligands have been developed to enhance drug release and circulation time.^4,5^ The conjugation of hydrophobic drugs and NPs with poly­(ethylene glycol) (PEG) is particularly well-established in cell surface engineering, as it enhances biocompatibility, water solubility, and dispersibility.^6^ NPs with PEGylated surfaces have a much longer *in vivo* circulation period because of the “stealth” quality they acquire.^7^ However, this same modification can also impede interactions between the nanoparticles and target cells, potentially reducing the efficiency of drug delivery.^8^

On the other hand, cholesterol (Chol) is a vital structural component of cell membranes and plays a key role in various biological processes, including the endocytosis of extracellular materials, as well as the proliferation and metastasis of cancer cells.^9^ Consequently, cholesterol has been copolymerized or conjugated with PLA-based systems to enhance drug encapsulation efficiency, improve cellular uptake, and maintain satisfactory hemocompatibility.^10–12^ Recently, PEG–cholesterol (PEG–Chol) has attracted significant attention among the several PEG conjugates. With its amphiphilic characteristics, the PEG–Chol molecule is made up of a hydrophilic PEG segment and a hydrophobic cholesterol attachment. This amphiphilicity imparts PEG–Chol molecules strong membrane-anchoring capabilities.^13,14^ Recently, we demonstrated the use of PEG–Chol as an anchor for lipids in developing a PLA-based biomimetic platform that incorporates functional transmembrane proteins.^15^

Breast cancer (BC) is a major global health concern, contributing significantly to the worldwide cancer burden. Cancer remains one of the leading causes of death globally, and BC is the most frequently diagnosed cancer in women.^16^ In 2022, there were 18.73 million new cancer cases and 9.67 million cancer-related deaths worldwide (excluding nonmelanoma skin cancer), underscoring the widespread impact of the disease. BC accounted for 6.9% of all cancer deaths, making it the fourth leading cause of cancer mortality overall and the leading cause of cancer deaths worldwide in women (15.4%).^17^ This high incidence across regions highlights the urgent need for improved prevention, early detection, and advanced treatment strategies to effectively reduce the global breast cancer burden.

Curcumin (Cur, 1,7-bis­(4-hydroxy-3-methoxyphenyl)-1,6-heptadiene-3,5-dione) and Tamoxifen (Tmx, 2-[4-(1,2-diphenylbut-1-enyl)­phenoxy]-*N*,*N*-dimethylethanamine) are well-established agents in modern BC therapy.^18^ Cur, a natural lipophilic compound extracted from the root of *Curcuma longa*, is widely recognized for its potent antioxidant, anti-inflammatory, and antitumor properties.^19–22^ Despite its therapeutic potential, Cur suffers from poor water solubility, which significantly reduces its bioavailability and leads to low absorption when administered orally.^22,23^ Similarly, Tmx, an estrogen receptor modulator,^24^ also exhibits very low water solubility but demonstrates good bioavailability through oral administration.^25^ Tmx is highly effective in reducing the recurrence of BC, lowering rates by approximately 50% within the first five years following diagnosis compared to nonendocrine treatments.^18^ However, extended use of Tmx is linked to considerable side effects. To optimize the therapeutic efficacy of both drugs while minimizing adverse effects, Cur and Tmx have been encapsulated in polymeric MPs and NPs, improving drug delivery and enhancing tumor targeting.^26–28^ Their lipophilic nature facilitates incorporation into nanocarriers via hydrophobic interactions, which increases the localized drug concentration at the tumor site.

Lately, there has been growing interest in utilizing highly porous materials for the treatment of BC, such as the zinc base zeolite imidazole skeleton material series (ZIF)^29^ and mesoporous silica NPs (MSNP).^30,31^ The enhanced porosity of these materials significantly improves drug delivery efficiency in BC treatment. Their key advantages include high porosity, large surface area, tunable pore sizes, and excellent biocompatibility. However, there remains ongoing debate regarding the low water stability and potential toxicity associated with ZIF-type carriers.^32^

The current work aims to synthesize novel PLA MPs with high porosity and amphiphilic PEG–Chol functionalization, thus combining the biocompatibility and low toxicity of PLA with the advantages of macroporous structures. The surface of drug-loaded PLA MPs was functionalized using a three-step protocol as shown in Figure 1.^15,33^ To the best of our knowledge, this represents the first report on functionalizing highly porous PLA particles with PEG-Chol for drug delivery applications. This functionalization was intended to improve particle stability in the bloodstream and enhance cell anchoring, promoting efficient endocytosis.

**Figure 1:**
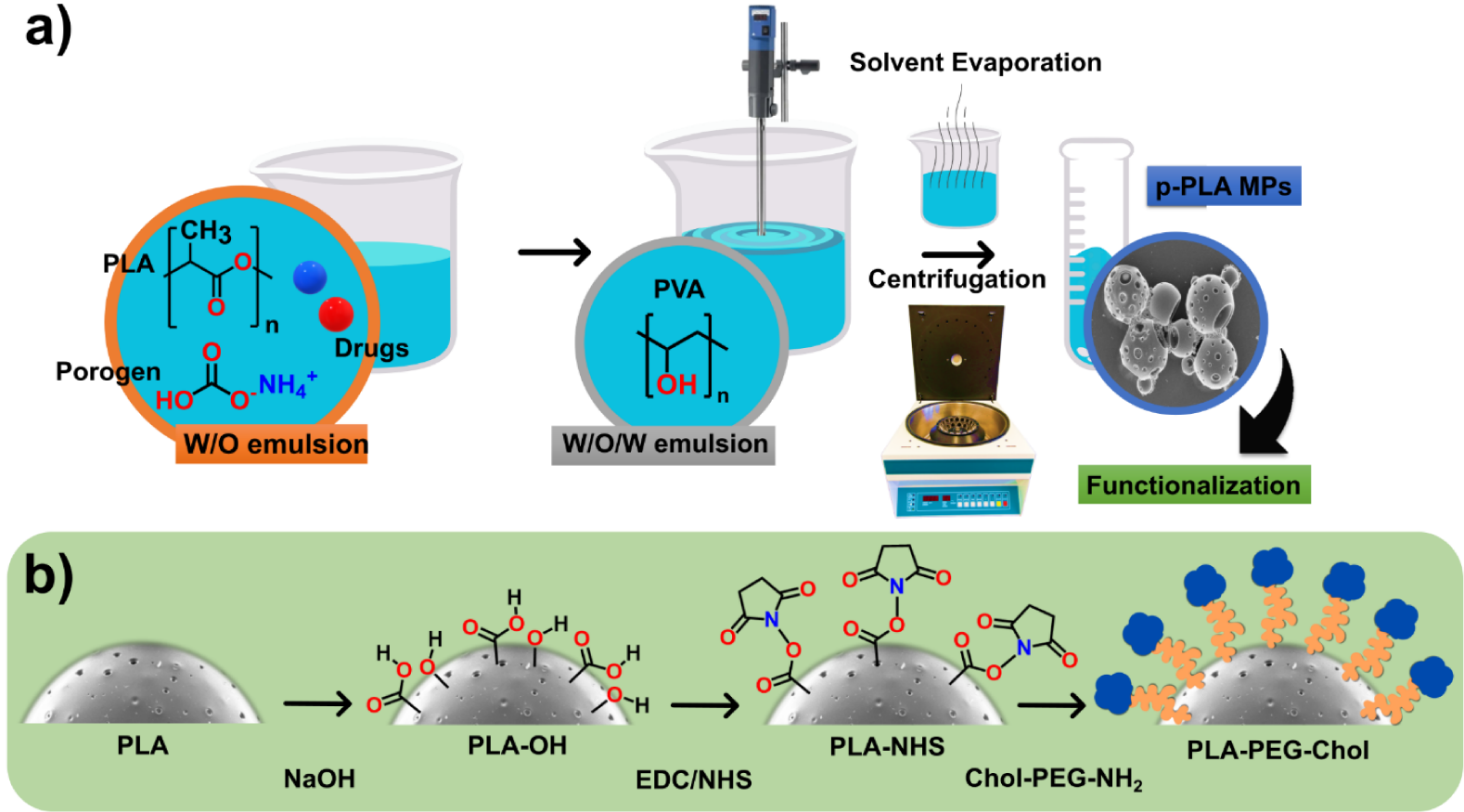
(a) A detailed description of the methodology used to obtain porous PLA microparticles (MPs) loaded with the drug, and (b) a three-step protocol for their surface functionalization.

To evaluate the functionality of this system, a proof-of-concept study was carried out to investigate its potential in BC therapy. The novel lipid-functionalized porous PLA MPs were loaded with the anticancer drugs Cur and Tmx to develop a hybrid drug delivery system for BC treatment. A cellular viability and uptake study was conducted to assess the potential of these particles for drug delivery, using the MCF-7 cell line as a model.^34^

## 2 Materials and Methods

### 2.1 Materials

Poly­(lactic acid) (PLA, *M*_n_ 98,100 g mol^–1^, *M*_w_ 181,000 g mol^–1^, and 1.85 of polydispersity), a NatureWorks product, was generously provided by Nupik International (Polinyà, Spain) (polymer 2002D). Poly­(vinyl alcohol) (PVA) (87–89% hydrolyzed, *M*_w_ 13,000–23,000 g mol^–1^), ammonium bicarbonate (NH_4_HCO_3_), tamoxifen (Tmx), curcumin (purity ≥65%, Cur), *N*-(3-(dimethylamino)­propyl)-*N′*-ethylcarbodiimide hydrochloride (EDC), *N*-hydroxysuccinimide (NHS), polyethylene glycol sorbitan monolaurate (Tween 20), dichloromethane (DCM), methanol (MeOH), phosphate buffered saline (PBS), 3-(4,5-dimethylthiazol-2-yl)-2,5-diphenyltetrazolium bromide (MTT), Hoechst 33258 dye, poly-l-lysine-FITC, and glutaraldehyde were obtained from Sigma-Aldrich (Spain). Chol-PEG-NH_2_ (purity: ≥95%) was purchased from Abbexa (UK). Human breast cancer cells (MCF7) and human normal prostate cells (PNT-2) were obtained from ATCC (American Type Culture Collection), Alexa Fluor 488 dye, CellTrace Calcein red-orange AM, Roswell Park Memorial Institute medium (RPMI), fetal bovine serum (FBS), trypsin-EDTA 0.25% phenol red, antibiotic/antimycotic solution (penicillin (100 units mL^–1^), streptomycin (100 μg mL^–1^), and amphotericin (25 μg mL^–1^), were purchased from ThermoFisher Scientific (Waltham, MA, USA).

### 2.2 Preparation of Drug-Loaded Porous PLA MPs

Drug-loaded porous PLA particles were generated by a modified emulsification-diffusion process.^35,36^ In summary, a total of 10 mg of the drugs Cur and Tmx were dissolved in a 2 mL solution containing 5% (w/v) PLA in DCM. The composition of the different components used is summarized in Table S1. To this polymer solution, a porogen solution consisting of NH_4_HCO_3_ (3%, 5%, and 10%; 1 mL) was added. The mixture was then subjected to sonication using a probe-type sonicator (Branson Digital Sonifier) for 60 s. As control, nonporous PLA particles were prepared by a similar method, without the addition of porogen–NH_4_HCO_3_ solution. In all formulations, the organic phase was introduced into a 6 mL aqueous solution of PVA (2%, w/v). The mixture was emulsified in an ice bath for 30 min at 10,000 rpm using an UltraTurrax (IKA, Staufen, Germany) and then mixed with 6 mL of a 1% (w/v) solution of PVA. The DCM was eliminated through agitation of the emulsion for 4 h at ambient temperature. Ultimately, the MPs were gathered using centrifugation at 10,000 rpm for 10 min (Eppendorf, Hamburg, Germany). Subsequently, the particles were rinsed twice with Milli-Q water before being used for subsequent procedures. The samples are denoted as p-PLA or n-PLA for porous and nonporous particles, respectively. Furthermore, when loaded with the drug, the porous and nonporous particles are designated as p-PLA–drug or n-PLA–drug, depending on whether they are loaded with Cur or Tmx, respectively.

In addition to the standard emulsification-diffusion process, individual experiments were conducted to evaluate the effect of each emulsification method on particle size and porosity. Specifically, particles were prepared using only the probe-type sonicator and, separately, using only the UltraTurrax homogenizer under the same conditions.

### 2.3 Functionalization of PLA MPs

In this study, we modified a previously published three-step protocol to facilitate the functionalization of PLA MPs with Chol–PEG–NH_2_.^33^ Initially, alkaline hydrolysis was employed to stimulate the surface of the MPs using a 0.1 M NaOH solution for 60 min. Following this, the particulates were washed, and recollected via centrifugation in order to eliminate the unreacted NaOH. In the subsequent phase, the particles were reconstituted in a 1 mL solution comprising a 1:1 ratio of EDC and NHS at their respective initial concentrations of 0.1 and 0.2 M. The precursor stock solution was prepared in a PBS solution with a pH of 7.4. The aforementioned technique is critical in the formation of amine reactive coupling groups on the PLA MPs’ surfaces. The reaction between EDC/NHS and the carboxyl groups produced on the surface of the polymer via the activation procedure utilizing NaOH resulted in the formation of NHS ester groups. Prior to the third phase, any residual NHS and EDC molecules were eliminated from the MPs by washing in Milli-Q water. Following this, the NHS moiety was utilized in the transamidation process to bond the Chol–PEG–NH_2_ building block with the activated surface of the PLA MPs. To facilitate the reaction, the PLA samples were subjected to a solution comprising Chol–PEG–NH_2_ at a concentration of 1 mg mL^–1^ in PBS for 4 h. As a result, amide connections were successfully formed. The samples were then rinsed to remove any unreacted molecules. In the interest of simplicity, the final product is designated as p-PLA–Chol–(Tmx or Cur).

### 2.4 Drug Entrapment Efficiency and Loading Capacity

The drug content was determined by dissolving lyophilized p-PLA–Tmx and p-PLA–Cur (10 mg mL^–1^ MPs) in 1.0 mL of a DCM:MeOH (1:1 v/v) solvent mixture. This solvent system ensures complete dissolution of the PLA matrix, thereby fully releasing the encapsulated drug into the medium. The suspension was sonicated and vortexed for 10 min to facilitate dissolution, after which it was centrifuged at 10,000 rpm for 10 min. Finally, the supernatant was evaluated using UV–vis spectroscopy. The calibration curve was prepared with the drug dissolved in DCM:MeOH, with the absorbance peak for Tmx and Cur read at 280 and 426 nm, respectively. The same procedure was applied to quantify the amount of the drug released during dialysis.

The entrapment efficiency (EE in %) and loading capacity (LC, in %) was calculated using the eqs 1 and 2, respectively.

1 $$\mathrm{EE}\;\%=\frac{\mathrm{Wt}\;\mathrm{of}\;\mathrm{Drug}\;\mathrm{Encapsulated}}{\mathrm{Total}\;\mathrm{Wt}\;\mathrm{of}\;\mathrm{Drug}\;\mathrm{Added}}\times 100$$

2 $$\mathrm{LC}\;\%=\frac{\mathrm{Wt}\;\mathrm{of}\;\mathrm{Drug}\;\mathrm{Encapsulated}}{\mathrm{Total}\;\mathrm{Wt}\;\mathrm{of}\;\mathrm{NPs}}\times 100$$

### 2.5 Characterization

The surface charge, size, and polydispersity of the drug-encapsulated MPs were assessed using a Zetasizer Nano ZS (Malvern Instruments Inc., Malvern, Worcestershire, UK). The chemical structures of PLA MPs containing the encapsulated drugs were verified by Fourier Transform Infrared (FTIR) spectroscopy. The successful functionalization of PLA MPs with PEG–Chol was validated by ^1^H NMR spectroscopy.

FTIR-ATR analysis was conducted using a FTIR Jasco 4100 spectrophotometer, equipped with an attenuated total reflection accessory (top-plate) and a Specac model MKII Golden Gate Heated Single Reflection Diamond ATR crystal. The instrument was linked to a computer running spectra management software (Spectra Manager) to observe the primary absorption bands of the PLA and drug compounds. Lyophilized materials were analyzed for absorption in the wavenumber range of 4000 to 600 cm^–1^. This analysis was conducted after 64 accumulation scans at a resolution of 8 cm^–1^ and baseline correction.

The ^1^H NMR spectra of PLA and PLA–PEG–Chol were acquired using a Bruker NMR Ascend 400 spectrometer operating at a frequency of 400 MHz. Each sample underwent 64 scans utilizing an 8 MHz sweep. The final spectra were analyzed using the TopSpin program.

Scanning electron microscopy (SEM) was used to investigate the morphology of the PLA MPs. The micrographs were acquired using a Zeiss Neon 40 scanning electron microscope equipped with a Focused Ion Beam, running at 2 kV. The samples were affixed to a carbon disc using double-sided tape and then coated, via sputtering, with a thin layer of conducting carbon to avoid electrical issues related to sample charging.

The thermal properties of the functionalized and drug-loaded MPs were analyzed using thermogravimetric analysis (TGA) and differential scanning calorimetry (DSC). TGA was performed using a PerkinElmer TGA 8000 instrument to determine the percentage of weight loss of the samples as a function of temperature in a controlled nitrogen atmosphere. Approximately 5 mg was placed in an aluminum pan and heated from 40 °C to 700 °C at a rate of 20 °C min^–1^. Thermal transitions of the samples were recorded using DSC with a PerkinElmer DSC7 instrument. The DSC measurements were carried out in the temperature range of 40 °C to 250 °C, with a heating rate of 10 °C min^–1^. Nitrogen (2 kg cm^–2^) was used as the purge gas.

### 2.6 Drug Release Kinetics

An in-house developed dialysis device containing 30 μL of p-PLA–Tmx, p-PLA–Chol–Tmx, p-PLA–Cur and p-PLA–Chol–Cur (10 mg mL^–1^ each) was utilized to evaluate drug release. The device was covered with a dialysis membrane of 6 kDa of molecular weight cutoff (MWCO) and submerged in 3 mL of PBS (pH 7.4) + Tween 20 (0.5%) release medium. The device was maintained in a shaker at 37 °C and 80 rpm. After the prescribed time period, in order to quantify the amount of drug released at the specific time stamp, the immersion medium was collected and substituted with 3 mL of fresh media. The experiment involved assessing the release process over a period of 7 days in a PBS solution. To facilitate the comparison of release kinetics, the total quantity of Tmx and Cur encapsulated within the MPs at the end of 7 days was extracted using the DCM-MeOH mixture to normalize the results. An assessment of the released drug quantity was conducted utilizing an UV–vis Cary 100 Bio spectrophotometer (Agilent, Santa Clara, CA, USA).

In order to generate calibration curves, the absorbance values obtained at 280 nm for Tmx and 426 nm for Cur were plotted against the drug concentration for two different solvent systems, i.e., PBS and DCM–MeOH, using free drugs. The mean of the drug release experiments conducted with a minimum of three replicates was graphed.

### 2.7 Cell Culture Maintenance

Human breast cancer cells (MCF7) and human normal prostate cells (PNT-2) were cultured in RPMI medium, supplemented with 10% FBS and 1% antibiotic/antimycotic solution, in T-flasks (75 cm^2^), at 37 °C and 5% CO_2_, with medium changed every 2 days. Cells were passaged at 90% confluence by incubation with trypsin–EDTA 0.25% solution for 3 min at 37 °C and subcultured at a 1:4 split ratio.

### 2.8 Cell Viability Assay

Cell viability assays were performed using the MTT assay following the protocol recommended by the manufacturer for 96-well plates.^37^ Briefly, cells were seeded at a concentration of 10 ^5^ cells mL^–1^ of medium in 96-well plates and incubated overnight at 37 °C and 5% CO_2_. The medium was replaced with fresh medium containing each type of drug or MPs, prepared by successive 1:2 dilutions starting from a 100 μg mL^–1^ solution. The cells were then incubated for 2 days at 37 °C in a 5% CO_2_ atmosphere. Following the incubation period, MTT labeling reagent was added to each well at a final concentration of 0.5 mg mL^–1^, and the cells were incubated for an additional 3 h under the same conditions. The solubilization agent (dimethyl sulfoxide) was then added to each well, and the plate was incubated for 15 min at 80 rpm. Finally, absorbance was measured at 570 nm using a microplate reader. The viability results correspond to the average of three independent replicas (*n* = 3) for each tested condition. Results were normalized to the control (without drugs or MPs addition), for relative percentages.

### 2.9 Cell Morphology Evaluation

The evaluation of cell morphology after exposure to each type of drug or MP was performed by confocal microscopy. Briefly, cells were seeded at a concentration of 10 ^6^ cells mL^–1^ of medium in 6-well plates, and incubated overnight at 37 °C and 5% CO_2_. The medium was then replaced by fresh medium supplemented with each type of drug or MP at concentrations of 50 μg mL^–1^, and in the absence of test condition (control). After this, cells were incubated for 2 days at 37 °C and 5% CO_2_. After the incubation period, cells were fixed using 2.5% glutaraldehyde solution in PBS, and stained with Alexa Fluor 488 phalloidin for visualizing actin cytoskeleton and with Hoechst dye to visualize the nuclei. In addition, for live cell assays, cells were stained with CellTrace calcein red-orange, and MPs were additionally functionalized with a fluorescent probe (poly-l-lysine-FITC). The amine groups of lysine interact with EDC/NHS analogous to NH_2_–PEG–Chol, facilitating covalent bonding to the MPs. This functionalization, owing to the fluorescence of FITC, enables the tracking of MPs by confocal microscopy. Samples were protected from light before imaging, which was performed using a 40× and a 63× objective of an Axio Observer 7 (Confocal laser microscope Carl ZEISS LSM 800). Imaging processing was completed with ZEN software and ImageJ software (Wayne Rasband, NIH, USA).

### 2.10 Colocalization of MPs within Cell Culture

The investigation of colocalization of MPs within the cells was performed by confocal microscopy. Briefly, cells were seeded at a concentration of 10 ^6^ cells mL^–1^ of medium in 6-well plates, and incubated overnight at 37 °C and 5% CO_2_. The medium was then replaced by fresh medium supplemented with MPs at a concentration of 50 μg mL^–1^, and in the absence of test condition (control). After this, cells were incubated for 2 days at 37 °C and 5% CO_2_. After the incubation period, cells were fixed using 2.5% glutaraldehyde solution in PBS, and triple stained with Alexa Fluor 488 phalloidin for visualizing actin cytoskeleton, with Hoechst dye to visualize the nuclei, and with CellTrace calcein red-orange to visualize the MPs since these can be cross-stained with this dye. Samples were protected from light before imaging, which was performed using a 63× objective of an Axio Observer 7 (Confocal laser microscope Carl ZEISS LSM 800). Imaging processing was completed with ZEN software.

## 3 Results and Discussion

### 3.1 Porous PLA MPs Fabrication

Bioresorbable porous PLA MPs loaded with antitumor agents Cur or Tmx were prepared using a modified water-in-oil-in-water (w/o/w) double emulsion method, following a modified version of a previously established procedure.^35,36^ In the fabrication process, NH_4_HCO_3_ was used as the porogen and added to the primary water phase. The organic phase consisted of PLA and the drugs dissolved in DCM, while the secondary water phase was composed of a 2% PVA aqueous solution. The incorporation of NH_4_HCO_3_ into the PLA MPs facilitates the formation of porous structures, as this inorganic material decomposes into NH_3_, CO_2_, and H_2_O. This decomposition creates a network of pores within the polymer core.^38^ Initially, the optimal amount of porogen was determined by studying MPs generated with 30%, 50%, and 100% (w/w) porogen, relative to the amount of PLA, corresponding to the p-PLA-30, p-PLA-50, and p-PLA-100 systems, respectively (Table S1). The porosity is beneficial because it helps to improve the bioresorption of the PLA matrix and enhances the release profile of the anticancer drugs that are encapsulated.^35^

SEM analysis was performed on the obtained MPs to evaluate the impact of porogen concentration on pore size, distribution, homogeneity, and the circularity and stability of the particles. Figure 2 illustrates the MPs obtained with different porogen concentrations, and Table S2 lists the measured particle and pore sizes obtained. As observed, when the concentration of NH_4_HCO_3_ is 30% (p-PLA-30), the p-PLA MPs exhibit a uniform distribution with well-defined pores. The average particle size is 1.3 ± 0.8 μm, and the pore size is 0.6 ± 0.3 μm. Increasing the porogen concentration to 50% NH_4_HCO_3_ results in larger pore diameters of 1.0 ± 0.4 μm, accompanied by a slight increase in particle size heterogeneity. However, at a concentration of 100% NH_4_HCO_3_ (p-PLA-100), significant particle collapse occurs, indicating a substantial compromise in structural integrity. The insets (Figure 2) offer a comprehensive perspective of the pore size distribution, highlighting the direct connection that exists between the concentration of porogens and the features of the pores that are present in the p-PLA MPs.

**Figure 2:**
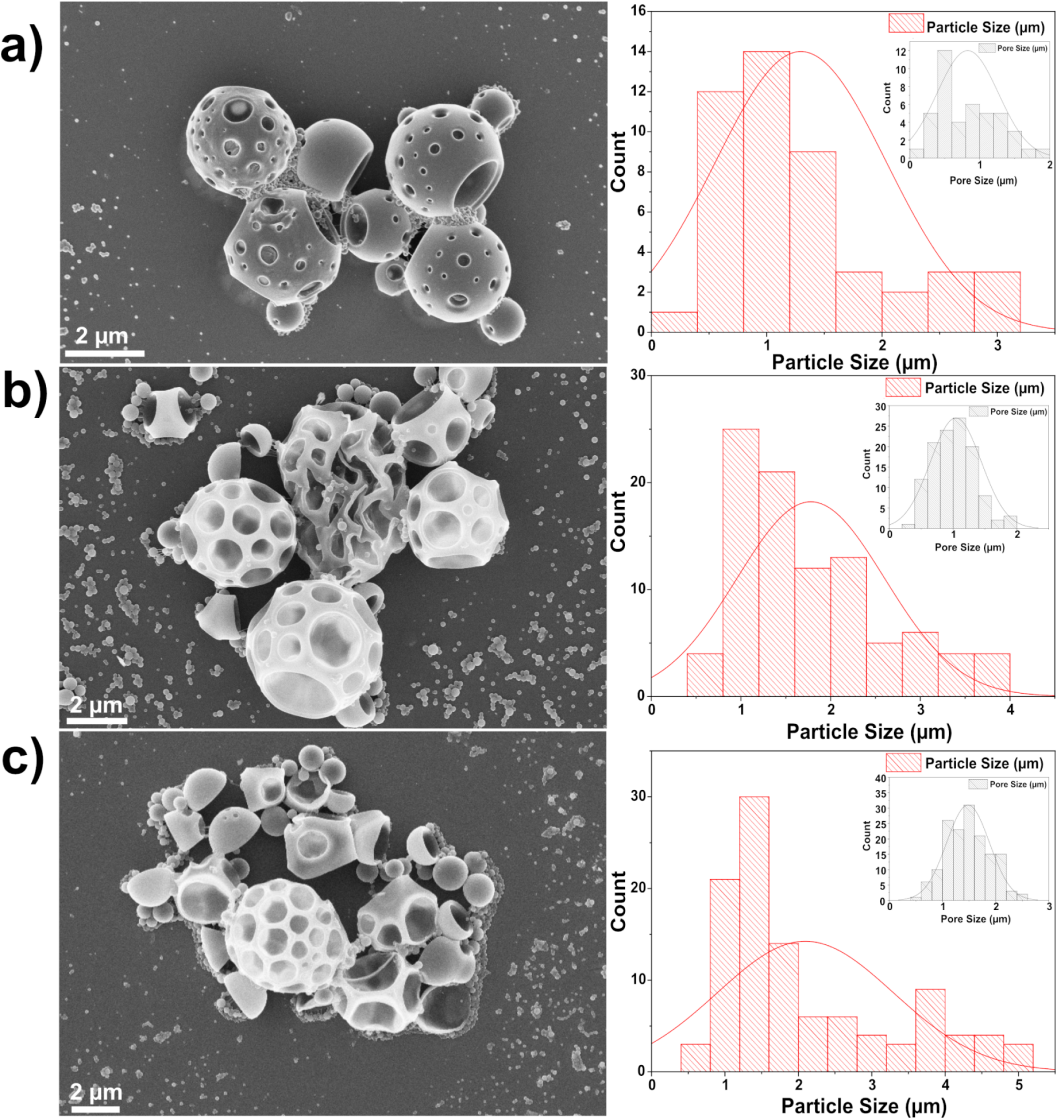
SEM images (left) and particle size distribution profiles (right) of p-PLA MPs synthesized with different concentrations of NH_4_HCO_3_ as the porogen: (a) 30%, (b) 50%, and (c) 100% (w/w) relative to PLA content. The insets in the right panel depict the corresponding pore size distributions of the MPs.

To further evaluate the effect of the emulsification steps on particle size and porosity, individual experiments were conducted using only the probe-type sonicator or only the UltraTurrax homogenizer. In the standard double emulsion (W/O/W) process, the primary emulsion was formed using the probe-type sonicator, which facilitates the incorporation of the porogen (NH_4_HCO_3_) within the polymer matrix. The secondary emulsion was then achieved using the UltraTurrax homogenizer to disperse the primary emulsion into the aqueous phase (Figure S1a–c). To assess the specific influence of each emulsification step, separate tests were performed where the primary emulsion was either directly emulsified into the aqueous phase using the UltraTurrax alone or generated solely by sonication without further homogenization (Figure S1b–d). The results indicated that sonication alone produced nanoparticles with minimal to no porosity, while UltraTurrax-processed particles exhibited few pores but with sizes exceeding 10 μm. These findings emphasize the necessity of both emulsification steps to achieve porous MPs with controlled size and morphology

Hence, a porogen concentration of 30% (w/w) was identified as optimal for drug loading (p-PLA-30), as MPs in the micrometer range are efficiently internalized by cells.^39^ Different cell lines have varying absorption limits for MPs, indicating the possibility of delivering cancer treatment drugs in a tailored manner.^40,41^ Additionally, due to their larger size, MPs are expected to have a higher drug loading capacity compared to NPs, which is particularly advantageous for cancer treatment. The enhanced drug loading capacity validates the effectiveness of delivering sufficient doses of anticancer drugs.

Functionalization of the MPs with Chol–PEG–NH_2_ was confirmed through ^1^H NMR analysis as it is depicted in Figure S2. The spectra of both pristine PLA and functionalized PLA–PEG–Chol particles, solubilized in CDCl_3_, were compared to assess the success of functionalization. In the PLA sample, two distinct proton sets were identified: one corresponding to the quadruplet of CH bonds and another to the doublet of CH_3_ groups. Figure S2 clearly demonstrates the presence of a CH_2_ peak at 3.65 ppm, primarily attributed to the PEG repeating units,^33^ confirming the successful attachment of PEG-Chol to the MPs.

Further confirmation of PLA functionalization with PEG–Chol was provided by SEM-EDX analysis. The SEM images (Figure 3) highlight the morphological features of PLA MPs both before and after functionalization with PEG–Chol. The prefunctionalization image (Figure 3a) shows the PLA MPs with a very smooth surface morphology. On the other hand, the postfunctionalization image (Figure 3b) reveals the formation of little globular aggregates on the MPs surfaces indicative of the successful PEG-Chol attachment.

**Figure 3:**
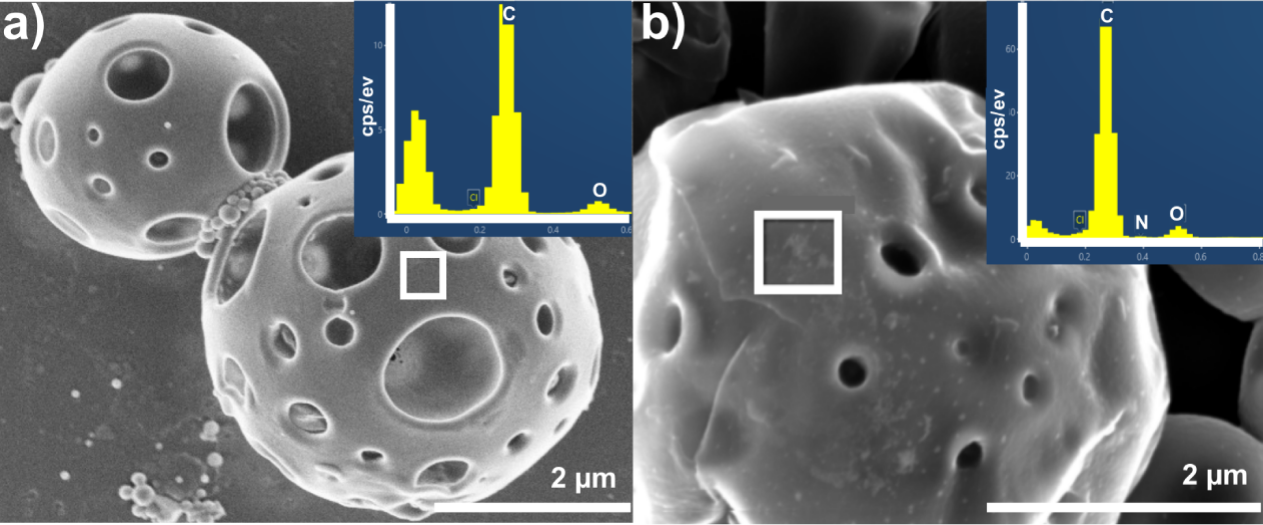
SEM images of PLA MPs a) before and b) after functionalization with PEG–Chol. Inset: EDX spectrum confirming the elemental composition PEG–Chol on the surface of particles, with prominent peaks corresponding to carbon (C), oxygen (O) and nitrogen (N). Micrographs recorded with 7000× magnifications are displayed.

The presence of PEG-Chol in these aggregates was also confirmed by the EDX spectrum (Figure 3b, inset). The EDX analysis of the globular aggregates on functionalized MPs revealed the presence of nitrogen (N) peaks, which were absent in the spectrum of nonfunctionalized MPs (Figure 3a, inset). The PLA matrix shows additional peaks corresponding to carbon (C) and oxygen (O), while the nitrogen signal specifically indicated the presence of PEG–Chol functional groups on the particle surface. The nitrogen atoms arise from the amide bonds present in PEG-Chol moieties.

### 3.2 Drug-Loaded MPs Characterization

p-PLA MPs were loaded with Cur and Tmx on account of their well-documented efficacy in treating cancer.^42,43^ FTIR spectroscopy was utilized to investigate the incorporation of the drugs into the MPs’ core, as shown in Figure 4. The main absorption bands of pure PLA, Cur, and p-PLA–Cur, p-PLA–Tmx are summarized in Table S3.

**Figure 4:**
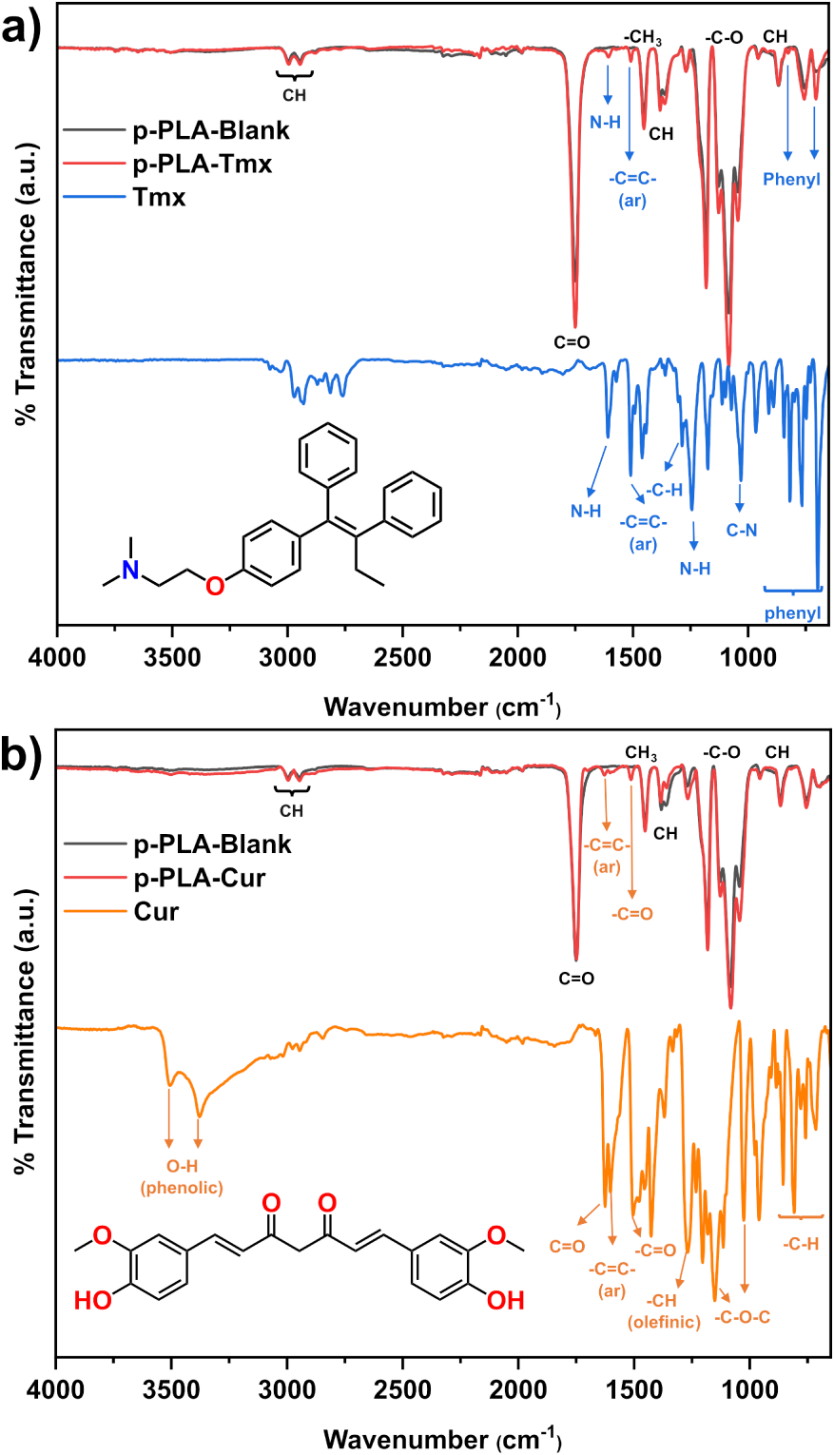
FTIR analysis comparing (a) p-PLA–Tmx MPs with pure Tmx and (b) p-PLA–Cur MPs with pure Cur. The spectra illustrate the key functional groups present in the MPs and their respective reference compounds, highlighting shifts in characteristic absorption bands that indicate interactions between the drugs and the PLA matrix.

The analysis of the FTIR spectra identified three distinct areas of PLA, consistent with the literature.^33^ The distinctive sharp peak at 1750 cm^–1^ represents the stretching of the carbonyl group (C=O) in the −CO–O– segment of PLA. Similarly, the sharp peak at 1182 cm^–1^ is caused by the stretching vibration of the −C–O– bond in the −CH–O– group of PLA polymer chains. Furthermore, the mountainous triplet peaks at 1128, 1085, and 1044 cm^–1^ are linked to C–O stretching vibrations in the ester groups within the polymer chains.

In the case of Tmx, the peaks observed at 1608 cm^–1^ and 1243 cm^–1^ are specifically associated with quaternary ammonium N–H stretching vibrations. The peaks observed at 1510 cm^–1^, 818 cm^–1^, 765 cm^–1^, and 698 cm^–1^ correspond to the stretching of different C–C bonds in the aromatic phenyl rings, which are critical components of the Tmx structure. The peak observed at 1286 cm^–1^ corresponds to the stretching of C–H bonds from the presence of terminal alkyl groups. Similarly, the peak at 1030 cm^–1^ indicates the stretching of amine C–N bonds. Upon loading the drug into PLA, three distinct peaks corresponding to Tmx were observed in the p-PLA-Tmx spectrum at 704 cm^–1^, 1509 cm^–1^, and 1606 cm^–1^. These peaks displayed a slight shift from their original positions. This displacement is attributed to weak physical interactions, including the formation of weak hydrogen bonds, van der Waals forces, dipole–dipole interactions, and similar phenomena.^44–46^

The FTIR spectra of Cur, PLA, and p-PLA–Cur, as outlined in Figure 4b, exhibit distinctive absorption bands specific to each component. The presence of phenolic O–H stretching vibrations is indicated by a wide absorption band observed at 3508 cm^–1^ and 3377 cm^–1^ in Cur. The prominent peak at 1626 cm^–1^ exhibits a primarily blended nature of C=O and C=C vibrations and a strong C=O vibration is seen at 1504 cm^–1^.^47^ In addition, the intense peak observed at 1600 cm^–1^ corresponds to the stretching vibration of the C=C bond within the aromatic benzene ring. Similarly, the peak at 1268 cm^–1^ indicates the stretching vibration of the olefinic C–H bond linked with the carbonyl group of Cur. The peaks observed at 1151 cm^–1^ and 1026 cm^–1^ are attributed to the stretching of the C–O–C bond, whereas the peak at 855 cm^–1^ is indicative of the aromatic C–H bonds. Moreover, the peaks observed at 959 cm^–1^ and 714 cm^–1^ correspond to the vibrations associated with the benzoate trans and cis C–H bonds.^48,49^

In p-PLA-Cur, the characteristic peaks of PLA are observed, along with two discrete peaks at 1512 cm^–1^ and 1627 cm^–1^, which show the presence of Cur in the polymer matrix. The absence of other peaks of drug moieties in both p-PLA–Cur and p-PLA–Tmx is likely due to substantial amount of absorption bands that coincide with those of PLA within the same wavenumber range. This is ascribed to the fact that the composite particles consist of a significant proportion of PLA. This suggests that, although Tmx and Cur are effectively incorporated into the PLA matrix, the dominant spectral features of PLA—due to its high proportion in the composite particles—mask the characteristic absorbance bands of the drugs, particularly in overlapping wavenumber regions.

In addition, the surface charge of different drug-loaded MPs was assessed through zeta potential measurements (ζ.). These analyses provided critical insights into the physical characteristics and colloidal stability of the MPs in a liquid state. UV–vis spectrophotometry was employed to quantitatively evaluate the entrapment efficiency (EE) and loading efficiency (LE) of the MPs, as defined in eqs 1 and 2. The results are summarized in Table 1.

**Table 1:** Comparative Analysis of PLA MPs Samples: Zeta Potential (ζ, in mV), Entrapment Efficiency (EE in %) and Loading Capacity (LC in %)

**Samples**	**ζ (mV)**	**EE (%)**	**LC (%)**
n-PLA	–20.9 ± 0.9	-	-
p-PLA	–33.5 ± 1.6	-	-
p-PLA–Cur	–28.1 ± 1.2	36.1 ± 2.1	3.3 ± 0.2
p-PLA–Tmx	–25.3 ± 0.9	59.2 ± 3.4	5.1 ± 0.3

Zeta potential measurements indicated that the surface charge of n-PLA MP was −20.9 ± 0.9 mV, while p-PLA had a surface charge of −33.5 ± 1.6 mV. These findings suggest that porous PLA particles exhibit greater stability compared to their nonporous counterparts. After drug entrapment, a noticeable alteration in the zeta potential was observed. This change can be attributed to the presence of hydrophobic drugs, with Tmx-loaded particles showing a more significant reduction in zeta potential because of its higher hydrophobicity. The zeta potential values were −28.1 ± 1.2 mV and −25.3 ± 0.9 mV for p-PLA–Cur and p-PLA–Tmx, respectively. The slight decrease in zeta potential is attributed to the formation of hydrogen bonds between the drug molecules and the PLA polymer chains.^50^ Overall, these values evidence the stability of the drug-loaded particles in suspension, which is crucial for their optimal performance in biomedical applications.

Finally, EE was found to be 36.1 ± 2.1% for p-PLA–Cur and 59.2 ± 3.4% for p-PLA–Tmx, with a corresponding LC of 3.3% and 5.1% for p-PLA–Cur and p-PLA–Tmx, respectively.

The encapsulation of 35 μg of Cur and 54 μg of Tmx per mg of PLA MPs in this study is particularly noteworthy, especially when compared to previous research. Although earlier studies have reported higher EE values, the drug loading per milligram of PLA MPs achieved here is substantially greater. The slightly lower EE, attributed to the highly porous structure of the polymer matrix, is offset by the significantly higher LC of the drug. In applications needing high payloads for focused therapeutic treatments, the potential of these MPs for effective drug delivery is underscored by this improved loading efficiency.^51,52^

### 3.3 Drug Release Kinetics

A preliminary kinetic study of drug release was conducted using both types of nonfunctionalized MPs—porous (p-PLA) and nonporous (n-PLA)—to investigate the impact of induced porosity on the release kinetics. Figure S3 illustrates a preliminary *in vitro* drug release study in PBS (pH 7.4) with 0.5% Tween over a 3-day period, with Cur as the encapsulated drug, while Figure S4 depicts the UV–vis calibration plots used to quantify the released Cur and Tmx. The data clearly demonstrate that p-PLA MPs exhibit significantly faster release kinetics compared to their nonporous counterparts, as expected. Specifically, p-PLA released over 40% of the encapsulated Cur after 72 h, whereas n-PLA MPs released only 17% of the total drug. Hence, we chose p-PLA for further investigation.

Furthermore, the influence of PEG-Chol functionalization on the drug release kinetics was examined, offering comprehensive insights into how this modification affects the release behavior of both drugs. Figure 5 illustrates the drug release kinetics of Cur and Tmx, both before (p-PLA) and after being modified with PEG–Chol (p-PLA–Chol). All formulations displayed the distinctive biphasic release pattern, characterized by an initial rapid release of drug molecules that were loosely attached to the surface of the particles, followed by a sustained release phase in which the remaining drug was gradually released from the core as a result of diffusion and the breakdown of the polymer matrix. Although no separate quantification of drug content before and after surface functionalization was performed, the preservation of the burst relase phase, particularly for Cur, strongly suggest that minimal drug loss occurred during the functionalization process. This observation is consistent with the assumption that surface-associated drug remained intact throughout the treatment. As the duration of the release extended, the curves exhibited a more linear profile, indicative of controlled release from the particle core.

**Figure 5:**
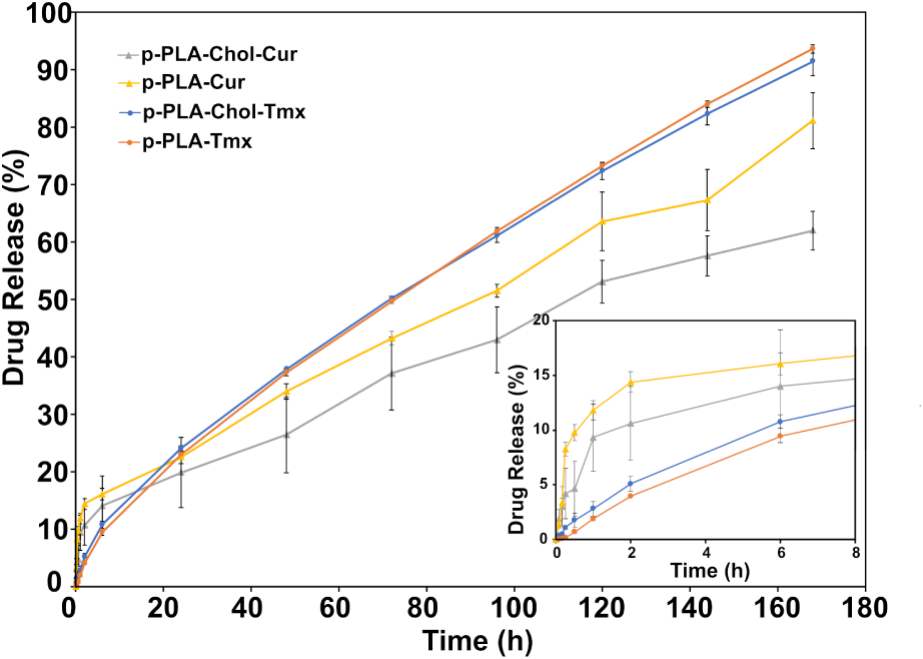
Drug release kinetics of nonfunctionalized p-PLA (p-PLA) and PEG–Chol functionalized p-PLA (p-PLA–Chol) MPs loaded with Tmx and Cur over a 7-day period in a PBS and 0.5% Tween mixture. The graph compares the % release profiles of Tmx and Cur from both types of MPs, emphasizing the influence of surface functionalization on drug release behavior. The inset highlights differences in the drug release during the initial hours.

Cur and Tmx interact differently with PLA and PEG-functionalized polymers, which affects their release profiles. Cur forms weak hydrogen bonds with PLA’s carbonyl groups via its hydroxyls, resulting in strong retention and limited diffusion-based release.^53^ In contrast, Tmx interacts with PLA mainly through dipolar interactions between its amine groups and the ester groups of the polymer, which are less retentive, allowing for easier release, especially when matrix erosion is involved.^54^ PEG, particularly when amphiphilically functionalized with cholesterol, improves water permeability and reduces Cur–PLA hydrogen bonding, thereby enhancing Cur release.^55^ Additionally, Tmx forms dual hydrogen bonding and hydrophobic interactions with PEG chains, creating stable complexes that enhance solubility and modulate diffusion-based release.^56^ These differences underscore the importance of designing drug delivery systems that align with each drug’s specific chemical behavior.

Indeed, notable differences in the sustained release patterns were observed among the formulations. The rapid release during the first 5 h was greater for both p-PLA–Cur and p-PLA–Chol–Cur compared to their Tmx-loaded counterparts. This may be attributed to the inadequate encapsulation of Cur (36.1%), leading to a higher amount of the drug being concentrated on the polymer’s surface and, thus, more easily diffusing into the release medium. In contrast, p-PLA–Tmx and p-PLA–Chol–Tmx showed better release patterns during the extended-release period, especially after 24 h. The improved release of Tmx in both formulations can be ascribed to its higher EE of 59.2%. This results in a more regulated and prolonged release from the particle core. These findings suggest that Cur’s interaction with the PLA matrix is strong, likely due to the formation of hydrogen bonds between the hydroxyl groups of Cur and the oxygen atoms in the ester linkages of PLA, which may hinder the diffusion of Cur from the polymer core.^57^ In contrast, the higher release rate of Tmx from the MPs indicates a simpler diffusion process, driven by the lower affinity between Tmx and the polymer matrix, resulting in a more efficient release of the drug from the core.

Within the same drug formulation, both p-PLA–Tmx and p-PLA–Chol–Tmx exhibited similar release profiles, suggesting that functionalization with PEG–Chol does not significantly influence the release kinetics of Tmx. In contrast, the functionalization of PLA with PEG–Chol had a marked impact on the sustained release phase of Cur. This effect is likely ascribed to the formation of hydrogen bonds and weak van der Waals interactions between Cur and the electronegative atoms in the PEG chains, as well as the hydrophobic cholesterol moieties, which probably modulate the drug release dynamics.

To better understand the mechanisms underlying the biphasic release profiles observed across all four formulations, the drug release data were fitted to established kinetic models commonly employed to describe release from polymeric matrices (Table S4 and Figure S5). These models included the Korsmeyer–Peppas model, which captures both Fickian diffusion and polymer matrix relaxation or erosion; the Higuchi model, which describes diffusion-controlled release from porous systems; and the Weibull model, an empirical function capable of characterizing both initial burst and sustained release phases. Fitting the experimental release profiles to these models allowed quantification of the relative contributions of diffusion, matrix relaxation, and formulation-specific variables—such as PEG–Chol functionalization—to the overall release behavior. Among the models, the Korsmeyer–Peppas model provided the best fit across all formulations (Figure S5a). The release exponent (*n*) values for Tamoxifen-loaded systems (PLA–Tmx: *n* = 0.73; PLA–Chol–Tmx: *n* = 0.69) indicated anomalous (non-Fickian) transport, suggesting that both diffusion and polymer relaxation or erosion processes contribute significantly to drug release. This finding is consistent with the physicochemical properties of PLA microparticles, particularly when modified with PEG–Chol, which likely enhances water uptake and matrix flexibility.^58^ In contrast, Cur-loaded systems—with and without PEG–Chol—exhibited *n* ≈ 0.51, indicative of Fickian diffusion, implying that passive diffusion through the PLA matrix was the dominant release mechanism. This behavior aligns with Curcumin’s low aqueous solubility and the high porosity of the polymer matrix.^59^ To further support this analysis, release data were also fitted to the Higuchi (Figure S5b) and Weibull (Figure S5c) models. The Higuchi model demonstrated strong linearity (adjusted *R* ^2^ > 0.96), reinforcing the conclusion that diffusion lead the sustained release phase. Meanwhile, the Weibull model effectively captured the overall release curve, showing the biphasic release behavior, distinguishing sigmoidal (*b* > 1) and burst-dominated (*b* < 1) profiles in Tamoxifen and Curcumin systems, respectively.

A stability assessment of blank and drug-loaded PLA–PEG–Chol MPs was performed in DMEM with fetal bovine serum. Blank MPs remained stable over 7 days, while drug-loaded MPs exhibited a gradual decrease in size after 3 days, consistent with drug release dynamics. Detailed results are presented in Figure S6.

Finally, thermal analysis was carried out to evaluate the thermal stability and drug dispersion of the different systems. TGA was performed on both drug-loaded and unloaded PLA–PEG–Chol MPs to evaluate their thermal stability, with comparisons made against the pure bulk PLA MPs (Figure S7a). Functionalization with PEG–Chol significantly enhanced the thermal stability of the pristine PLA MPs, which otherwise exhibited the most pronounced weight loss. Likewise, the incorporation Cur and Tmx further improved the thermal stability of the functionalized MPs. Analysis of the first derivative of the thermograms (Figure S7b) revealed an increase of approximately 10 °C in the PLA MPs degradation temperature upon PEG-Chol functionalization. In contrast, drug loading with Tmx and Cur resulted in additional increases of 3 °C and 13 °C, respectively, compared to the functionalized MPs, indicating further stabilization due to drug incorporation.

DSC analyses were conducted on functionalized and drug-loaded MPs and compared with pristine PLA (Table S5 and Figure S8). For Tmx-loaded MPs, a decrease of approximately 4 °C in the glass transition temperature (*T*_g_), accompanied by a slight increase in the heat capacity change (ΔC_p_^m^), was observed. These findings indicate a mild plasticizing effect, likely due to the incorporation of the drug and the presence of PEG functional groups, which enhance chain mobility and increase the amorphous fraction relative to pristine PLA. This interpretation is further supported by the more pronounced cold crystallization peak observed in Tmx-loaded MPs. A similar, albeit less pronounced, effect was detected in Cur-loaded MPs. The observed increase in the amorphous phase and slight disruption of crystalline order may also explain the reduction in the melting temperature (*T*_m_) of the drug-loaded systems. These thermal transitions suggest that the drug molecules are well incorporated within the polymer matrix, likely at the molecular level. Overall, the data are consistent with a homogeneous dispersion of the drug within the PLA-based MPs.

### 3.4 Cell Viability Study

After establishing the release profiles of MPs loaded with Tmx or Cur, the impact of each particle type and the corresponding free drug on cell viability and morphology was evaluated. This assessment was conducted on cultured cells after a 2-day exposure period. MCF-7 cells, a model for human BC, were used for this analysis, while PNT-2 cells were used as a control as representative of normal human cells

Starting with normal human cells, cell viability remained above 80% under all tested conditions (Figure 6, bottom left panel), with two exceptions. For p-PLA–Chol–Tmx MPs at a concentration of 100 μg mL^–1^, viability dropped to 70%. More notably, exposure to free Cur led to a significant decrease in cell viability: at 50 μg mL^–1^, viability reduced to 50%, and further decreased to approximately 10% at 100 μg mL^–1^. These results agree with previously reported cell viability values showing that normal cells experience reduced viability at Cur concentrations of 50 μg mL^–1^ and higher.^60^

**Figure 6:**
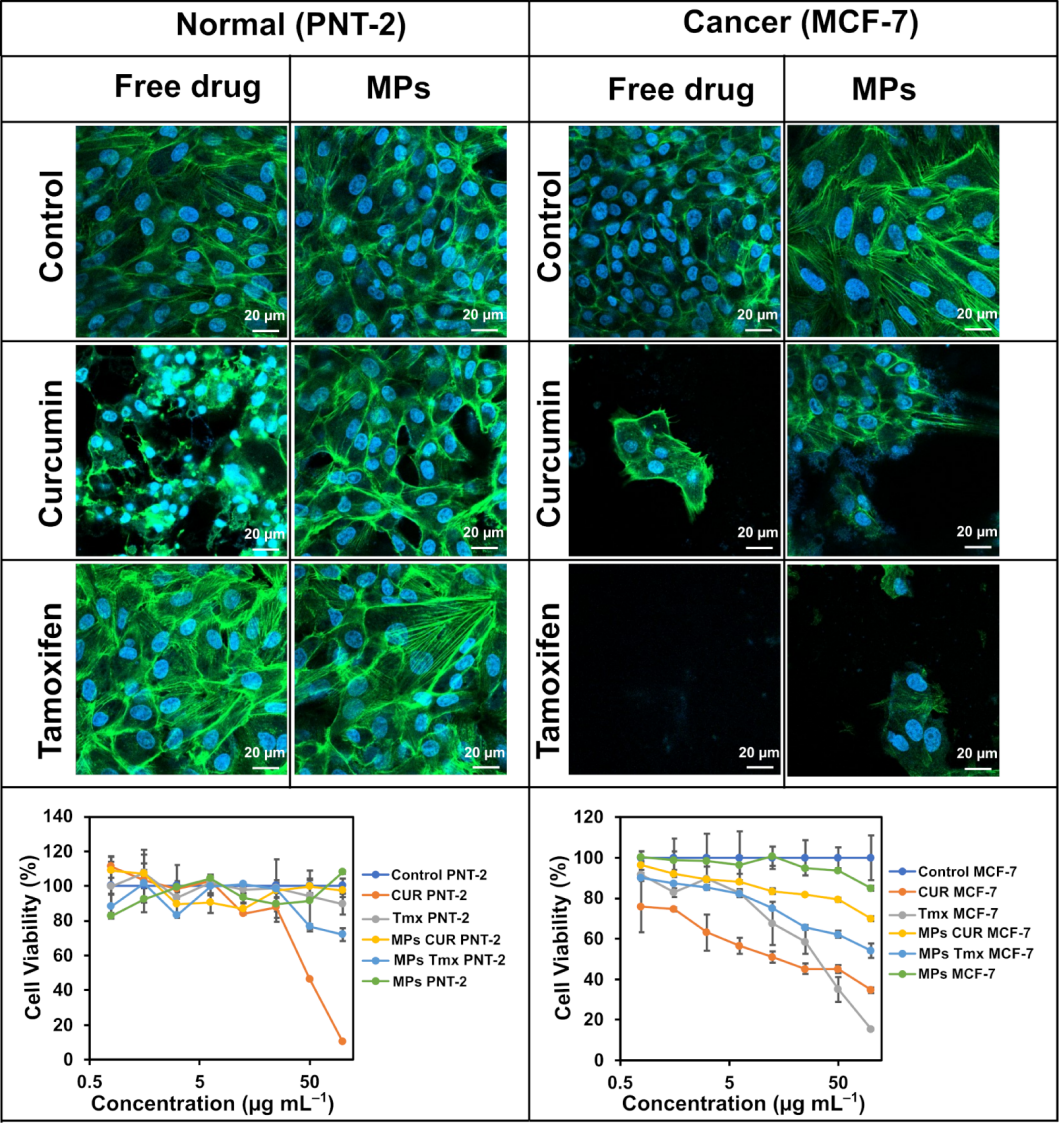
Top panel: Confocal microscopy images of human normal prostate cells (PNT-2, left) and BC cells (MCF-7, right) incubated with p-PLA–Chol–Tmx and p-PLA–Chol–Cur MPs, and respective free drug solutions at a concentration of 50 μg mL^–1^, for 2 days. Green fluorescence: actin filaments; blue fluorescence: cell nuclei. Bottom panel: Cell viability curves of human normal prostate cells (PNT-2) and BC cells (MCF-7) exposed to varying concentrations of p-PLA–Chol–Tmx and p-PLA–Chol–Cur MPs, and respective free drug solutions, for 2 days.

It is noteworthy that the local release of Cur from p-PLA–Chol–Cur MPs does not reach concentrations high enough to compromise the cell viability, likely due to its slower release kinetics and distinct intracellular cytotoxic profile compared to Tmx. Indeed, even at the highest concentrations of these MPs, cell viability remained nearly 100%. These findings are consistent with observations from confocal microscopy after 2 days of exposure to p-PLA–Chol–Tmx and p-PLA–Chol–Cur MPs, as well as the corresponding free drugs at a concentration of 50 μg mL^–1^. The microscopy images revealed normal nuclear morphology and a high expression of actin, indicated by the abundance of highly organized, linear green actin filaments distributed throughout the cell cytoplasm in all tested conditions, except for free Cur (Figure 6, top left panel). At 50 μg mL^–1^ of Cur, normal cells showed nuclei with a slightly necrotic appearance and actin filaments were more disorganized and less expressed by the cells. Most importantly, given the overall results, both p-PLA–Chol–Tmx and p-PLA–Chol–Cur MPs are safe to be used with normal human cells, not having significant impact on cell viability or morphology.

In contrast, when examining the effects of p-PLA–Chol–Tmx and p-PLA–Chol–Cur MPs, along with their respective anticancer drugs in solution, on BC cells, both drugs demonstrated high cytotoxicity (Figure 6, bottom right panel).

The cell viability of MCF-7 cells exposed to the pure drugs in solution showed the expected increase in cytotoxicity. As the concentration of Cur in solution increased, MCF-7 cell viability steadily decreased, reaching approximately 30% at the highest concentration. Similarly, exposure to Tmx in solution resulted in a significant decrease in breast cancer cells viability, with MCF-7 cells showing only 10% viability at 100 μg mL^–1^ of Tmx, suggesting that this drug has a stronger anticancer effect when compared to Cur.

The same trend was followed by p-PLA–Chol–Cur and p-PLA–Chol–Tmx MPs. However, although both decreased cancer cell viability, the effect of p-PLA–Chol–Tmx MPs was superior to that of p-PLA–Chol–Cur MPs. Indeed, at the highest concentrations of MPs, the BC cell viabilities were around 70% for p-PLA–Chol–Cur MPs, and as low as 50% for p-PLA–Chol–Tmx MPs. These results were supported by the observation of the MCF-7 cells by confocal microscopy (Figure 6 top right panel). The significant cytotoxic effect of 50 μg mL^–1^ Tmx was evident, as no cells remained in culture after 2 days of exposure to the drug. Similarly, 50 μg mL^–1^ Cur also reduced cell count, although not as significant as for Tmx. Both types of p-PLA–Chol MPs demonstrated a reduction in breast cancer cell count, with p-PLA–Chol–Tmx MPs showing a more substantial impact. These findings align with previous reports in literature, where Tmx is recognized as a widely used and effective cancer treatment, with successful results documented in several clinical trials.^61–64^ Cur, while known for its cytotoxic effects in different cancer types,^57,60^ is also noted as a promising coadjuvant drug to be used in cancer treatment,^65–67^ particularly for its synergistic effects when combined with Tmx.^68^

### 3.5 Cellular Uptake of MPs

Confocal microscopy was utilized to show the uptake of MPs by tumor cells, with a comparison to human normal cells, to gain insights into the role taken by p-PLA–Chol MPs in drug transport via endocytosis. Specifically, both normal and BC cells were incubated for 2 days with p-PLA-Chol MPs functionalized with FITC, imparting green fluorescence to the MPs. Meanwhile, live cells were stained with Calcein Red-Orange AM, producing red fluorescence, as illustrated in Figure 7.

**Figure 7:**
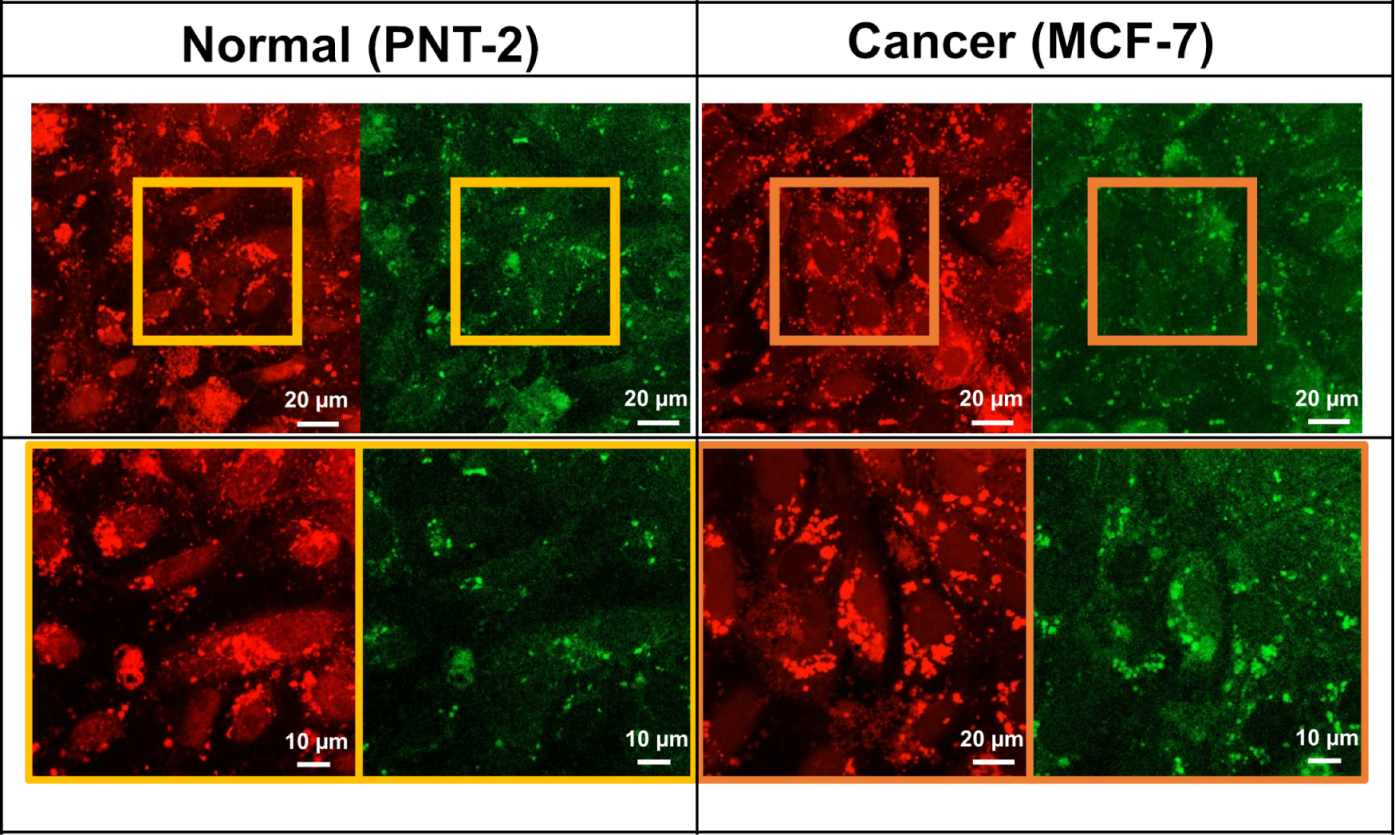
Confocal microscopy images of live human normal prostate cells (PNT-2) and BC cells (MCF-7) stained with Calcein Red-Orange AM (red), and incubated with p-PLA–Chol MPs functionalized with FITC (green) for 2 days.

The red fluorescence intensity observed through confocal microscopy indicates that both cell lines remained viable after incubation with MPs, as confirmed by cell viability assays (Figure 6), where nearly 100% viability was observed for both cell lines without drug loading. Notably, this technique also enabled indirect visualization of the cell nucleus shape. When comparing these micrographs with those showing green fluorescence from the FITC functionalized p-PLA–Chol MPs, an accumulation of MPs around the nuclei in both cell types was observed. Hence, not only do MPs interact with the cell membranes of normal and BC cells, but are also internalized by the cell and accumulate near the nucleus.

To assess the cellular uptake of the MPs and its colocalization within the cell, additional confocal microscopy experiments were conducted using both cancerous and normal cells in a triple staining method. The images (Figure 8) confirm that, despite their relatively large size, MPs are effectively internalized and localized near the nucleus. This observation was validated by triple staining of the actin cytoskeleton, nuclei, and MPs, with individual channel images provided in the Supporting Information (Figure S9). The lateral views demonstrate that the MPs are completely enclosed within the cytoplasm rather than merely adhering to the cell membrane. Given their size, the uptake mechanism is likely driven by macropinocytosis or phagocytosis, processes that involve actin-dependent membrane remodeling. The 48-h incubation period ensured sufficient time for internalization, supporting the feasibility of MPs as intracellular delivery vehicles.

**Figure 8:**
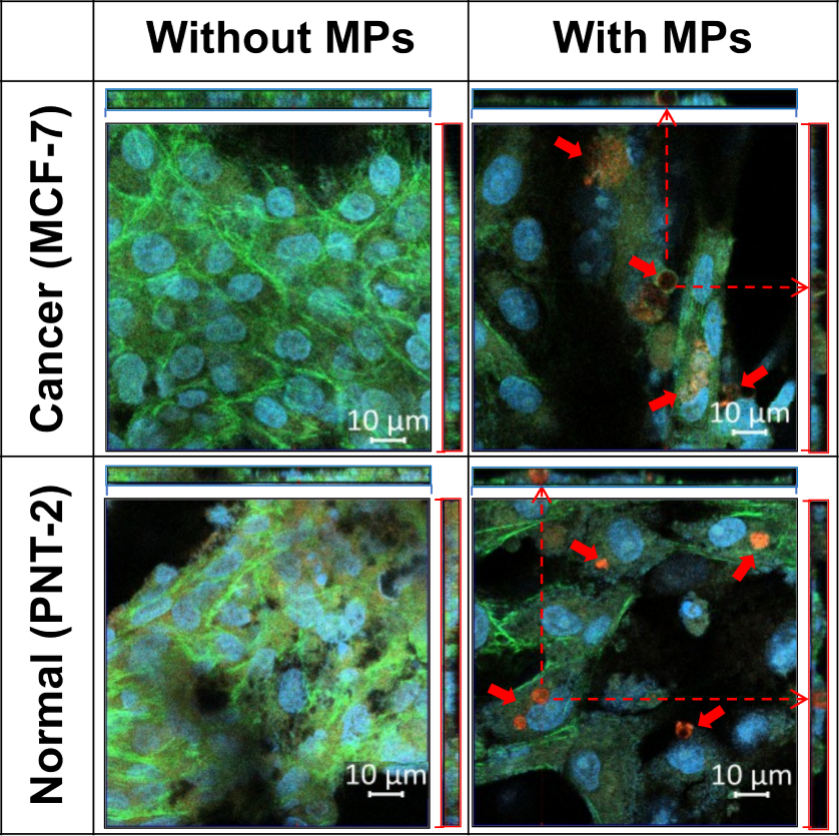
Confocal microscopy images of live human normal prostate cells (PNT-2) and breast cancer cells (MCF-7), triple stained with Alexa Fluor 488 phalloidin for actin cytoskeleton visualization, Hoechst dye for nuclei staining, and CellTrace calcein red-orange for MP detection.

Confocal microscopy results of p-PLA–Chol MPs within both cell types indicates a high efficiency of MPs uptake, consistent with previously reported results for PEDOT NPs in breast and prostate cancer therapy.^57,60^ Moreover, regarding the diameter of the p-PLA–Chol MPs, they are well-suited for tumor accumulation and cellular uptake, as drug carriers with sizes up to 5 μm can be internalized by cells through phagocytosis, macropinocytosis, or receptor-mediated endocytosis, mechanism further facilitated by targeting anchors like PEG–Chol.^69–72^ In this regard, several studies have demonstrated that particle uptake significantly enhances drug delivery efficacy, particularly when integrated into therapeutic systems, highlighting the importance of material design in optimizing therapeutic outcomes.^73–76^

To complement the qualitative confocal microscopy observations, a quantitative analysis of MPs uptake was performed using image-based quantification. The number of internalized functionalized MPs was assessed in both PNT-2 and MCF-7 cells using ImageJ software, based on confocal images (Figure S10). Interestingly, this analysis confirmed enhanced uptake in MCF-7 cells compared with normal cells.

Overall, the release of the anticancer drugs from p-PLA–Chol–Tmx and p-PLA–Chol–Cur MPs is most likely to occur within BC cells rather than in the surrounding tumor, suggesting that a localized therapy could possibly be accomplished.

## 4 Conclusions

Engineered carriers with surface modifications are crucial for effective drug encapsulation. In this study, porous PLA MPs were fabricated using an optimal 30% NH_4_HCO_3_ concentration, resulting in well-defined pores and stable structures that enable efficient drug loading, positioning them as promising drug carriers. Further functionalization with PEG-Chol improved surface characteristics, as confirmed by SEM-EDX and ^1^H NMR analyses. Drug loading studies revealed significant entrapment of Cur and Tmx, with enhanced loading efficiency compared to previous research. Preliminary kinetic studies demonstrated a faster drug release from porous MPs, especially for Cur, while functionalization with PEG–Chol modulated release dynamics. Cell viability assays confirmed the safety of p-PLA–Chol MPs on normal cells and their effective cytotoxicity against BC cells, with Tmx showing superior anticancer effects. These results highlight the potential of p-PLA–Chol MPs as effective delivery vehicles for cancer therapy.

## Supplementary Material



## Notes

Fabrication parameters of MPs, averaged particle and pore size, identification peaks from FTIR spectra, SEM images of MPs, ^1^H NMR spectra of functionalized MPs, drug release rates between porous and nonporous particles, UV–vis calibration curves, kinetic modeling of drug release profiles, degradability studies of MPs, TGA and DSC thermograms, confocal microscopy images, and quantitative analysis of MPs internalization of live human normal prostate and BC cells (PDF)
