# Supplementary material for: Cholesterol-Functionalized Porous PLA Microparticles for Enhanced Drug Delivery
Source: ACS Appl Bio Mater. 2025 Sep 4;8(9):7743–56. doi: 10.1021/acsabm.5c00693 (PMC12464973; doi:10.1021/acsabm.5c00693)
Supplement: Supplementary file 1 [file mt5c00693_si_001.pdf]

Supporting Information

Cholesterol-Functionalized Porous PLA Microparticles for Enhanced Drug Delivery

Ahammed HM Mohammed-Sadhakathullah,[1,2] Leonor Resina,[1,2] Hamidreza Enshaei, [1,2] Kristina Ivanova,[3] Tzanko Tzanov,[3] Maria M. Pérez-Madrigal, [1,2] Elaine Armelin,[1,2] and Juan Torras[1,2,]*

[1] Innovation in Materials and Molecular Engineering – Biomaterials for Regenerative Therapies (IMEM-BRT) Group, Departament d'Enginyeria Química, EEBE, Universitat Politècnica de Catalunya, C/ Eduard Maristany 10-14, Building I, 2nd floor, 08019 Barcelona, Spain

[2] Barcelona Research Center for Multiscale Science and Engineering (BRCMSE), Universitat Politècnica de Catalunya, C/ Eduard Maristany, 10-14, Building I, basement floor, 08019, Barcelona, Spain.

[3] Grup de Biotecnologia Molecular i Industrial, Department of Chemical Engineering, Universitat Politècnica de Catalunya, Rambla Sant Nebridi 22, Terrassa 08222, Spain.

*Corresponding author: joan.torras@upc.edu

Table S1. Overview of the various fabrication parameters involved in the manufacturing of MPs. This includes the composition of the polymer matrix, the type and concentration of the porogen agents, and anti-tumor agents used.

Reactants	PLA	NH_4HCO_3	Curcumin	Tamoxifen
n-PLA	100 mg	-	-	-
p-PLA-30	100 mg	30 mg	10 mg	10 mg
p-PLA-50	100 mg	50 mg	-	-
p-PLA-100	100 mg	100 mg	-	-

Table S2. Averaged particle and pore size (in μm) of PLA with increasing porogen concentration.

	**Size**	
	Particle	Pore
p-PLA-30	1.3 ± 0.8	0.6 ± 0.3
p-PLA-50	1.8 ± 0.8	1.0 ± 0.4
p-PLA-100	2.1 ± 1.1	1.5 ± 0.4

Table S3. Identified peaks in FTIR spectra of PLA MPs loaded with curcumin (p-PLA-Cur) and tamoxifen (p-PLA-Tmx), referenced against pure curcumin (Cur) and tamoxifen (Tmx), respectively.

Sample	Wavenumber (cm^{-1})	Functional groups
PLA [1]	2995, 2946	−CH−
	1750	C=O (ester)
	1452	$-CH_3$
	1381, 1362	−CH−
	1269, 1182, 1128, 1085, 1044	C−O−C (ester)
	868, 756	−CH
Tmx [2-4]	1608, 1243	N−H
	1510	−C=C− (aromatic)
	1286	−C−H
	1030	−C−N−
Cur [5-7]	3508, 3377	−O−H (phenolic)
	1624, 1504	−C=O
	1600	−C=C− (aromatic)
	1268	−C−H (olefinic)
	1151, 1026	−C−O−C
	855	−C−H (aromatic)
	959, 714	−C−H

Table S4. Summary of kinetic model parameters fitted to drug release profiles from PLA-based MPs loaded with Tmx or Cur, with or without PEG–Chol functionalization. Parameters from Higuchi (diffusion constant k_H), Korsmeyer–Peppas (release rate constant k and exponent n), and Weibull (scale a and shape b) models are reported along with adjusted R^2 values indicating goodness of fit.

Models	Korsemeyer-Peppas $F(t) = k \cdot t^n$			Higuchi $F(t) = k_H \cdot \sqrt{t}$		Weibull $F(t) = 1 - exp(-(\frac{t}{a}) b)$		
**Sample**	**k**	**n**	**R^2**	**k**	**R^2**	**a**	**b**	**R^2**
PLA-Tmx	2.2040	0.7315	0.9997	6.5264	0.9744	91.0779	1.1598	0.9915
PLA-Chol-Tmx	2.6648	0.6891	0.9996	6.4605	0.9815	92.1739	1.0694	0.9909
PLA-Cur	5.3645	0.5113	0.9669	5.6531	0.9692	128.4306	0.6745	0.9436
PLA-Chol-Cur	4.5132	0.5053	0.9812	4.6262	0.9825	210.1071	0.6232	0.9694

Table S5. Glass transition temperature (T_g, ºC), specific heat capacity change at T_g (ΔC_p^g, J ºC^{-1} g^{-1}), cold crystallization peak temperature (T_c, ºC), melting peak temperature (T_m, ºC), and specific melting enthalpy (ΔH_m, J g^{-1}) of functionalized PLA-based MPs loaded with Tmx or Cur, compared with pristine PLA. Standard error is also shown.

	**T_g**	**ΔC_p^g**	**T_c**	**T_m**	**ΔH_m**
PLA	60.3±1.3	0.30±0.02	73.2±0.0	152.7±0.4	31.2±0.2
PLA-PEG-Chol-Tmx	56.2±0.4	0.32±0.06	95.4±0.6	151.8±0.8	26.0±0.9
PLA-PEG-Chol-Cur	59.7±0.3	0.29±0.02	101.3±0.5	148.8±0.3	26.3±0.2

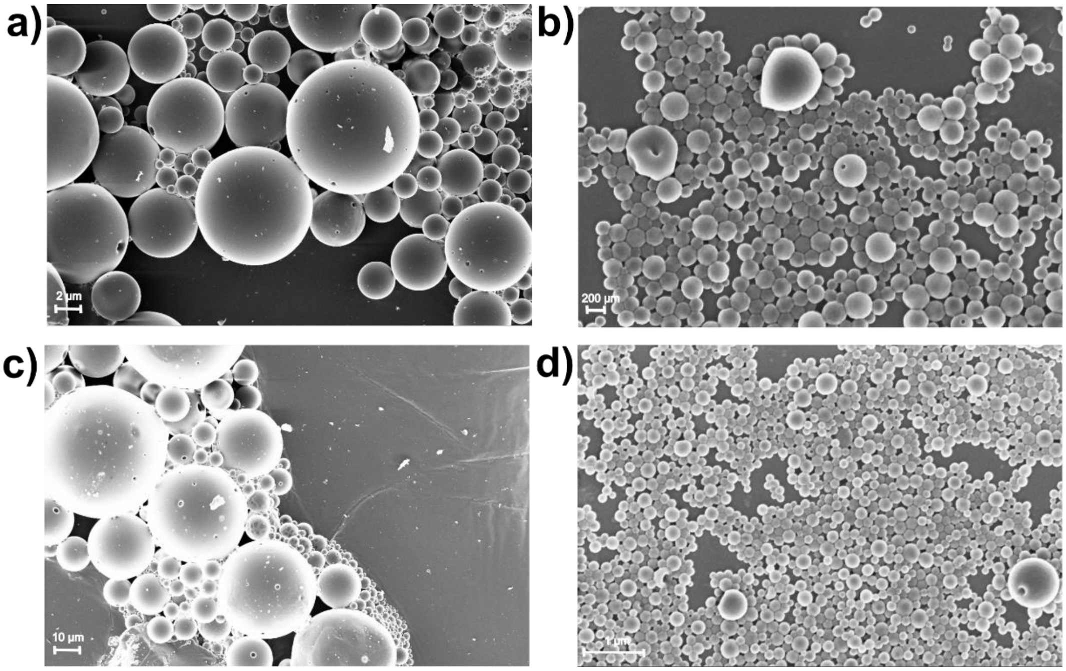

Figure S1. SEM images of p-PLA MPs at two different magnifications, a) and c) synthesized using only the UltraTurrax® homogenizer (left) and, b) and d) only the probe-type sonicator (right).

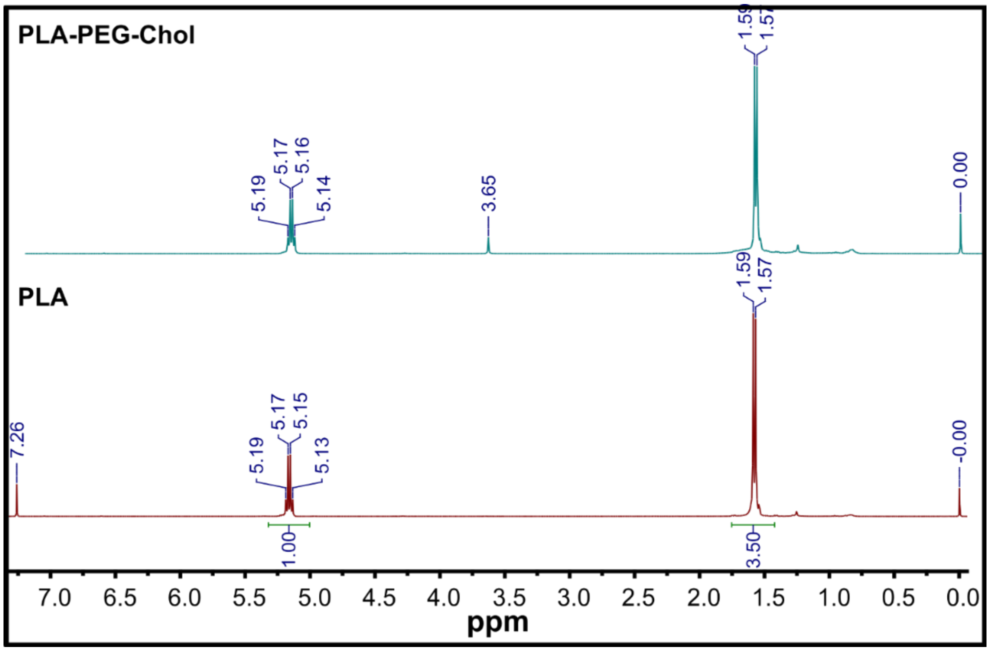

Figure S2. ^{1}H-NMR spectra of PLA and PLA-PEG-Chol functionalized samples, illustrating chemical shifts and structural modifications.

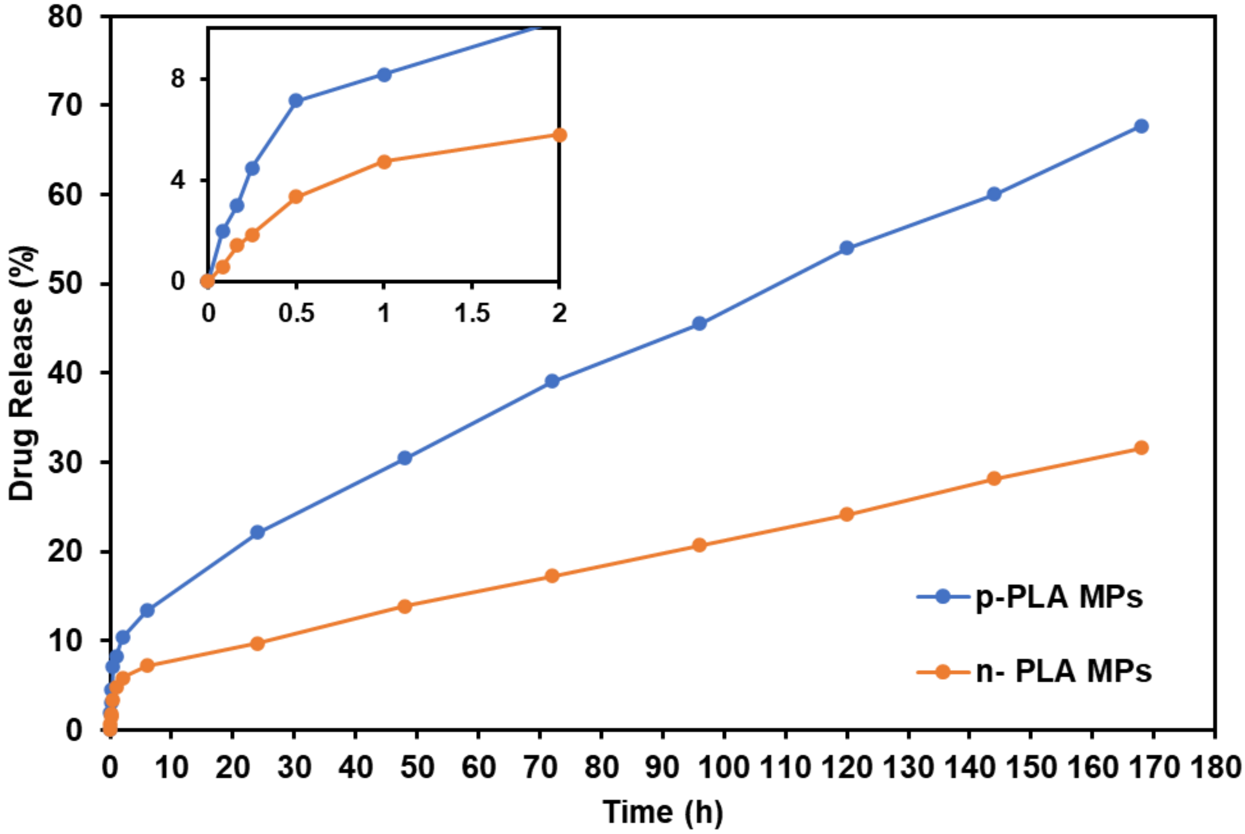

Figure S3. Drug release rates between porous (p-PLA) and non-porous (n-PLA) MPs. The release profiles demonstrate the gradual release of the enclosed drugs over time, offering insights into the varying release patterns of the two MP formulations.

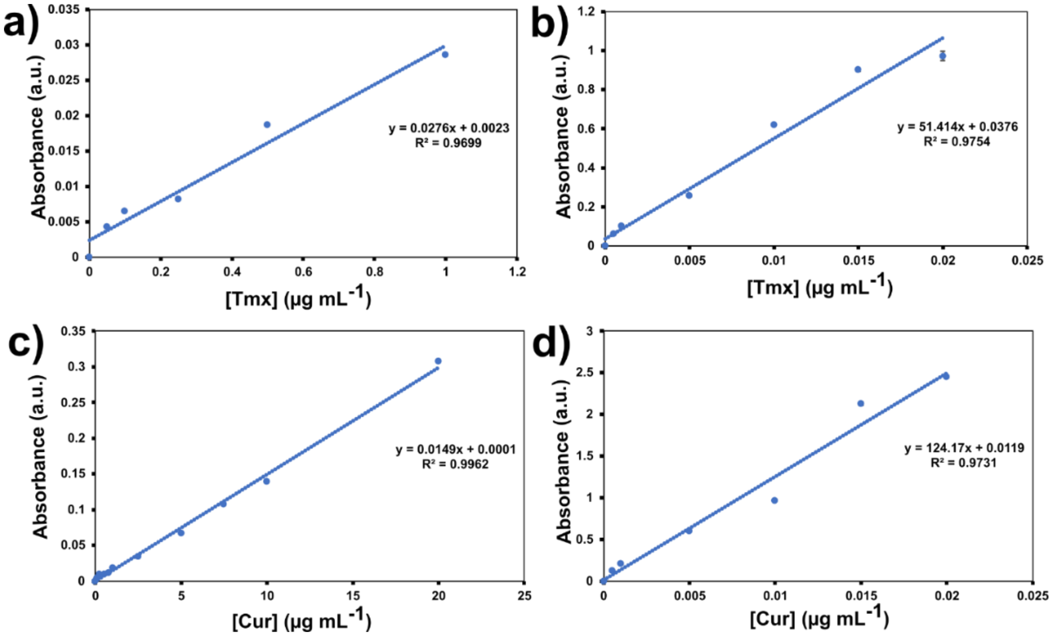

Figure S4. Calibration curves for drug quantification in two different solvent systems used for release and extraction studies. a) and c) represent the calibration curves generated in PBS with 0.5% Tween, utilized for *in vitro* drug release studies. Curves b) and d) correspond to the calibration curves in DCM-MeOH solvent mixture, applied for drug extraction efficiency.

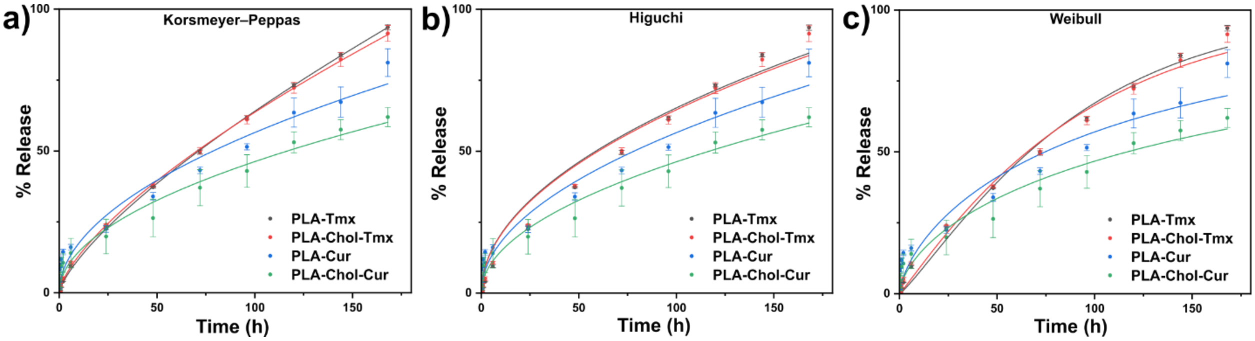

Figure S5. Kinetic modeling of drug release profiles from PLA-based MPs formulations. Release data for four formulations (PLA-Tmx, PLA-Chol-Tmx, PLA-Cur, and PLA-Chol-Cur) were fitted to (a) Korsmeyer–Peppas, (b) Higuchi, and (c) Weibull models. Each panel displays the experimental cumulative release data overlaid with the corresponding model-fitted curves.

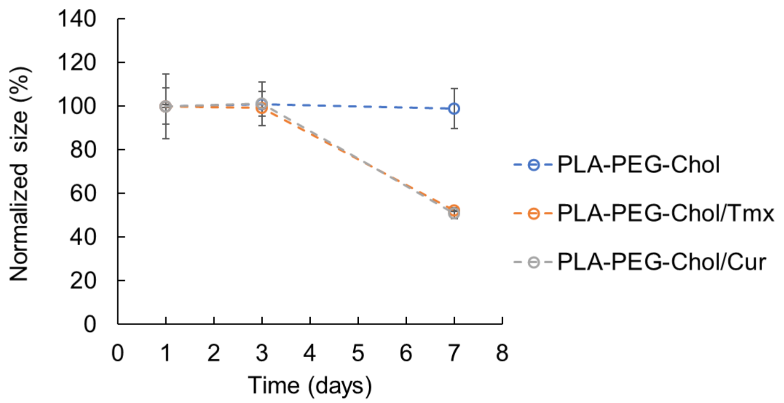

Figure S6. Stability evaluation of PLA-PEG-Chol, PLA-PEG-Chol-Tmx, and PLA-PEG-Chol-Cur MPs in DMEM medium supplemented with fetal bovine serum (FBS) over a period of 7 days. Hydrodynamic diameter was monitored to assess particle integrity and potential degradation or drug release over time.

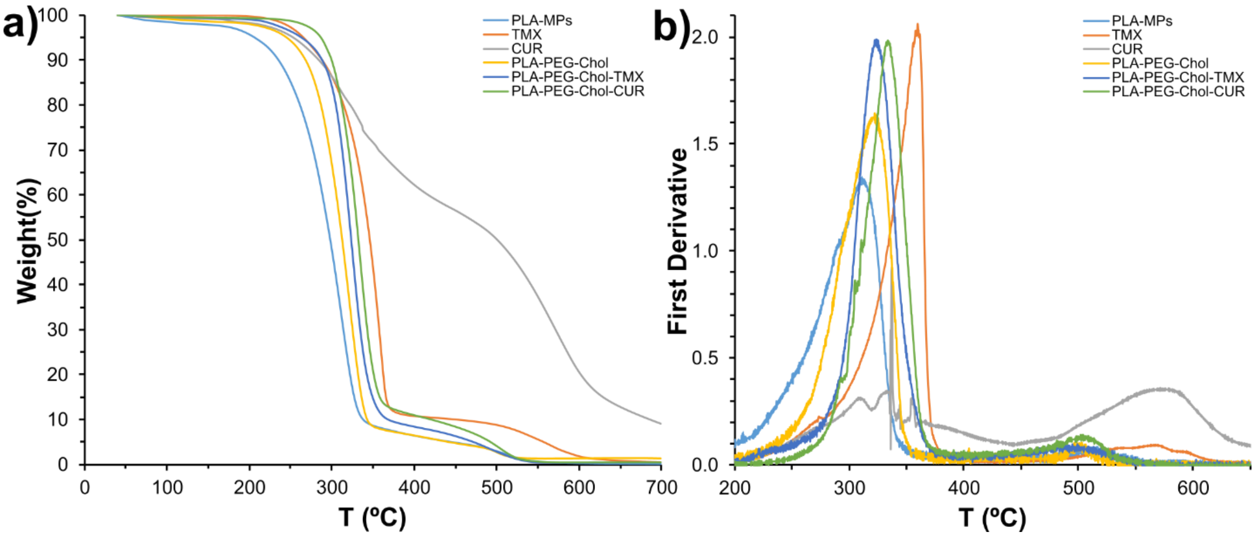

Figure S7. (a) Thermogravimetric analysis (TGA) and (b) first derivative thermogravimetric curves of PLA-based MPs. All curves showing the thermal degradation profiles of PLA-MPs, PLA-PEG-Chol MPs, and drug-loaded MPs (PLA-PEG-Chol-Tmx and PLA-PEG-Chol-Cur).

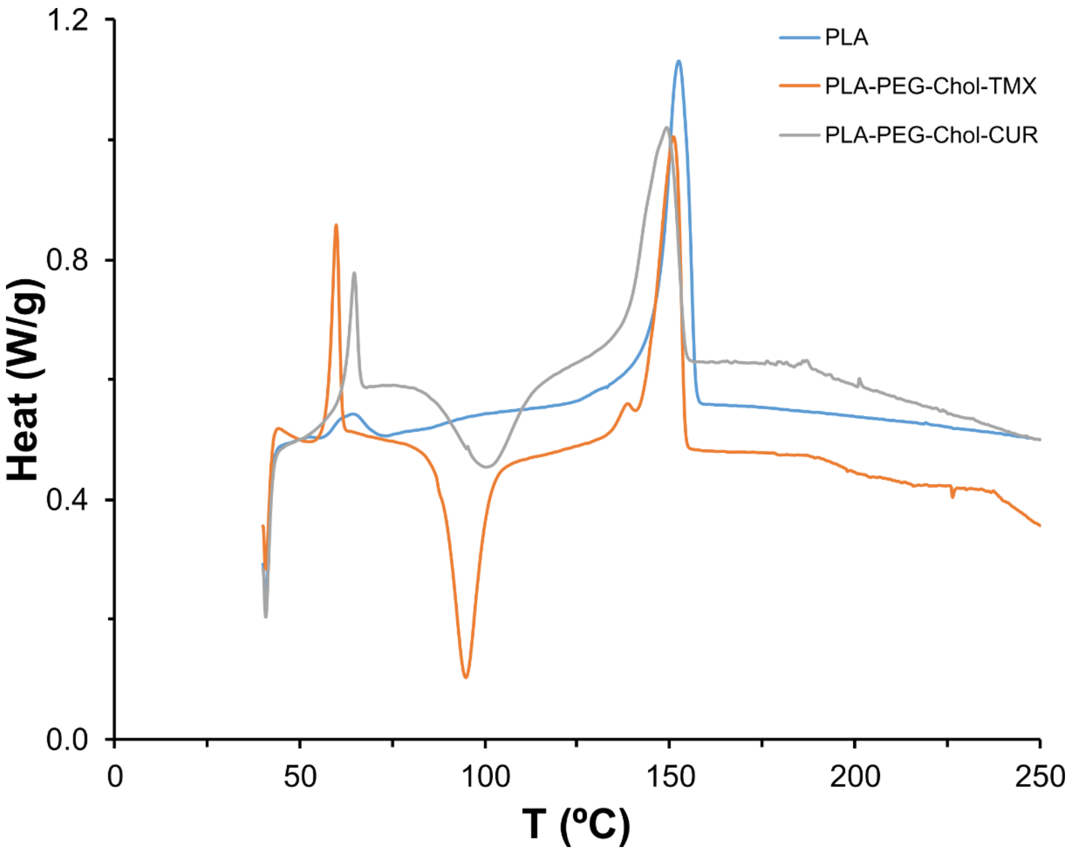

Figure S8. Representative DSC thermograms of pristine PLA and drug-loaded MPs (PLA-PEG-Chol-Tmx and PLA-PEG-Chol-Cur) recorded at a heating rate of 10 °C min^{-1}. The curves show a glass transition around 60 °C, followed by cold crystallization and subsequent melting. Endothermic transitions are oriented in the upward direction.

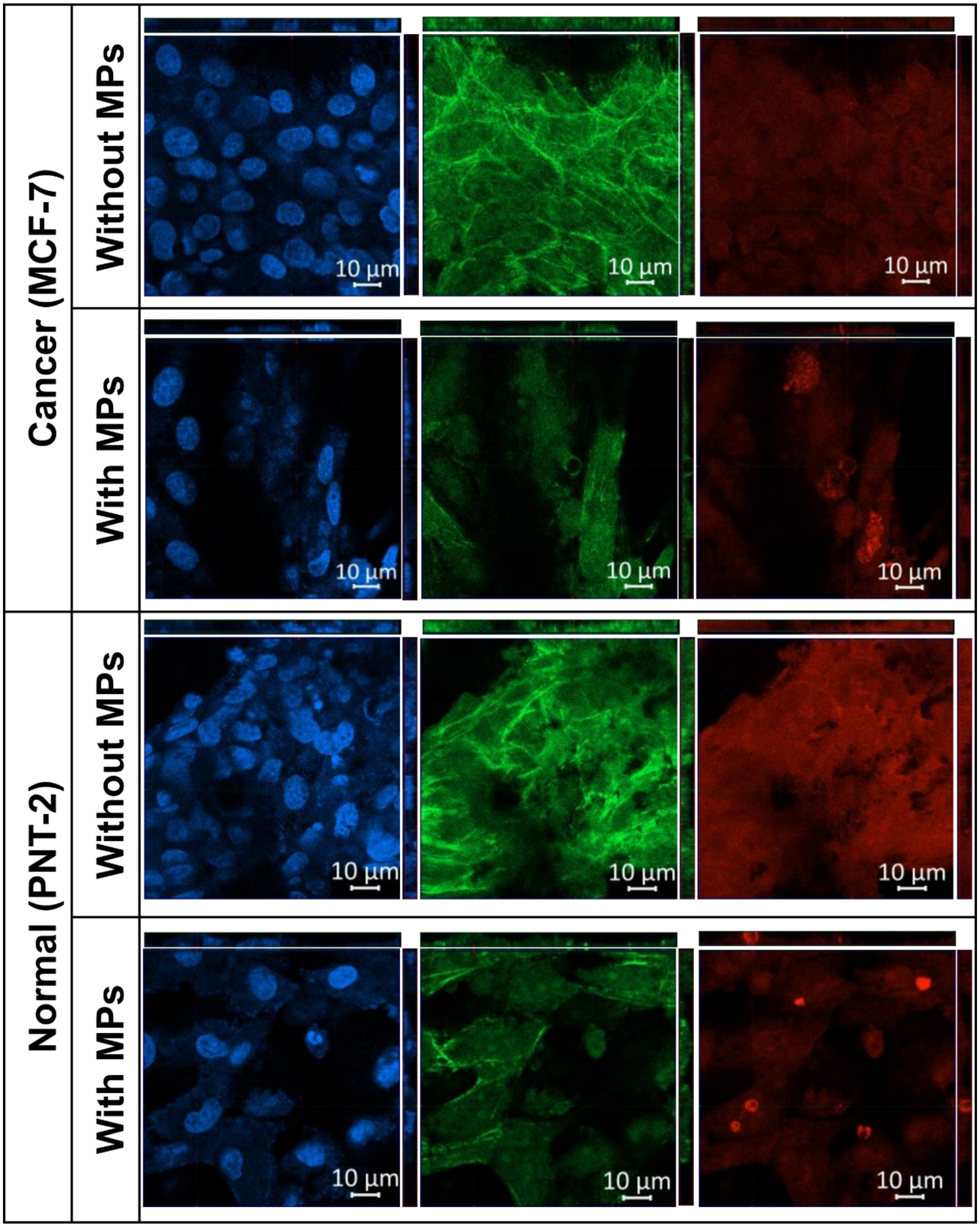

Figure S9. Confocal microscopy images of live human normal prostate cells (PNT-2) and BC cells (MCF-7), triple stained with Hoechst dye to visualize the nuclei, with Alexa Fluor 488 phalloidin for visualizing actin cytoskeleton, and with CellTrace™ calcein red-orange to visualize the MPs. The three channels are shown in blue, green and red, respectively.

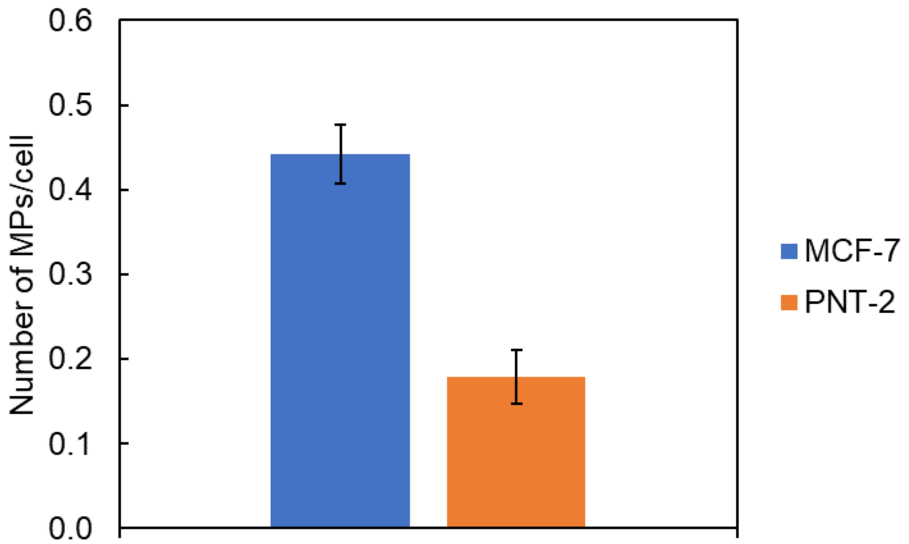

Figure S10. Quantitative analysis of MPs internalization in normal prostate epithelial cells (PNT-2) and breast cancer cells (MCF-7). The number of internalized MPs per cell was determined from confocal microscopy images using ImageJ software.

REFERENCES

(1) Mohammed-Sadhakathullah, A. H. M.; Paulo-Mirasol, S.; Molina, B. G.; Torras, J.; Armelin, E. PLA-PEG-Cholesterol biomimetic membrane for electrochemical sensing of antioxidants. *Electrochim. Acta* **2024**, *476*, 143716. DOI: https://doi.org/10.1016/j.electacta.2023.143716.

(2) Maji, R.; Dey, N.; Satapathy, B.; Mukherjee, B.; Mondal, S. Preparation and characterization of Tamoxifen citrate loaded nanoparticles for breast cancer therapy. *Int. J. Nanomedicine* **2014**, *9*, 3107-3118. DOI: https://doi.org/10.2147/IJN.S63535.

(3) Liu, Z.-P.; Zhang, Y.-Y.; Yu, D.-G.; Wu, D.; Li, H.-L. Fabrication of sustained-release zein nanoparticles via modified coaxial electrospraying. *Chem. Eng. J.* **2018**, *334*, 807-816. DOI: https://doi.org/10.1016/j.cej.2017.10.098.

(4) Li, J.-J.; Yang, Y.-Y.; Yu, D.-G.; Du, Q.; Yang, X.-L. Fast dissolving drug delivery membrane based on the ultra-thin shell of electrospun core-shell nanofibers. *Eur. J. Pharm. Sci.* **2018**, *122*, 195-204. DOI: https://doi.org/10.1016/j.ejps.2018.07.002.

(5) Kolev, T. M.; Velcheva, E. A.; Stamboliyska, B. A.; Spiteller, M. DFT and experimental studies of the structure and vibrational spectra of curcumin. *Int. J. Quantum Chem.* **2005**, *102* (6), 1069-1079. DOI: https://doi.org/10.1002/qua.20469.

(6) Yu, J. Y.; Kim, J. A.; Joung, H. J.; Ko, J. A.; Park, H. J. Preparation and characterization of curcumin solid dispersion using HPMC. *J. Food Sci.* **2020**, *85* (11), 3866-3873. DOI: https://doi.org/10.1111/1750-3841.15489.

(7) Mohan, P. R. K.; Sreelakshmi, G.; Muraleedharan, C. V.; Joseph, R. Water soluble complexes of curcumin with cyclodextrins: Characterization by FT-Raman spectroscopy. *Vib. Spectrosc.* **2012**, *62*, 77-84. DOI: https://doi.org/10.1016/j.vibspec.2012.05.002.
